# The Contribution of Genetic Modifiers to Ovarian Cancer Risk in *BRCA1* and *BRCA2* Pathogenic Variant Carriers

**DOI:** 10.3390/cancers18030354

**Published:** 2026-01-23

**Authors:** Dagmara Cylwik, Roksana Dwornik, Katarzyna Białkowska

**Affiliations:** Department of Genetics and Pathology, International Hereditary Cancer Center, Pomeranian Medical University, 72-204 Szczecin, Poland

**Keywords:** *BRCA1*, *BRCA2*, ovarian cancer risk, genetic modifier

## Abstract

The article presents the current state of knowledge on genetic modifiers of ovarian cancer risk in women carrying pathogenic variants (PVs) in the *BRCA1* and *BRCA2* genes, which are major contributors to hereditary susceptibility to this malignancy. Although PV carriers have high disease penetrance (*BRCA1*: ~40% and *BRCA2*: 11–27%), substantial variability in individual risk is observed, suggesting the influence of additional genetic variants. **Background:** Ovarian cancer is characterized by late detection and high mortality, and a significant portion of risk among *BRCA1/2* carriers is shaped by reproductive and environmental factors as well as genetic modifiers. The article emphasizes that carriers of the same *BRCA* PV can exhibit markedly different risk levels depending on additional variants that modulate key biological processes, such as DNA repair, cell cycle regulation, and apoptosis. **Methods:** A systematic literature search covering the years 1996–2025 was conducted in the PubMed database. Initially, 734 publications were identified; after removing duplicates, thematically irrelevant articles, non-full-text papers, and studies not meeting the inclusion criteria, 47 articles were included in the review. These studies covered candidate gene analyses, GWAS, and data from the CIMBA consortium, which enables the examination of large cohorts of PV carriers. **Results:** The review identified numerous variants associated with increased or decreased ovarian cancer risk in *BRCA1* carriers, including the following: *OGG1*, *DR4*, *MDM2*, *CYP2A7*, *CASP8*, *ITGB3*, *HRAS1*, *TRIM61*, and *MTHFR.* The reviewed studies also identified both protective and risk-increasing variants among *BRCA2* PV carriers: *UNG*, *TDG*, and *PARP2*, and haplotypes in *ATM*, *BRIP1*, *BARD1*, *MRE11*, *RAD51*, and *9p22.2.* The analysis identified 11 variants affecting both *BRCA1* and *BRCA2* carriers, most of which increase risk, including the following: *IRS1*, *RSPO1*, *SYNPO2*, *BABAM1*, *MRPL34*, *PLEKHM1*, and *TIPARP.* Protective variants include *BNC2* and *LINC00824*. The only SNP reaching genome-wide significance (*p* < 5 × 10^−8^) was in *BNC2*. **Conclusions:** The article summarizes the growing number of genetic modifiers of ovarian cancer risk among *BRCA1/2* carriers and highlights their potential to improve individualized risk assessment, enhance patient stratification, support personalized prevention and surveillance strategies, deepen the understanding of disease biology, and identify potential therapeutic targets.

## Simple Summary

Women who carry pathogenic variants in the *BRCA1* or *BRCA2* genes have a high risk of developing ovarian cancer, but this risk varies greatly between individuals. This variability cannot be explained by *BRCA1/2* variants alone and suggests that other genetic factors play an important role. This review summarizes current evidence on additional genetic variants, known as genetic modifiers, which can increase or decrease ovarian cancer risk in *BRCA1/2* carriers. Based on a systematic review of studies published between 1996 and 2025, including candidate gene studies and large genome-wide association analyses, multiple risk-modifying variants were identified for *BRCA1* carriers, *BRCA2* carriers, and both. Most of these variants influence biological pathways involved in DNA repair, cell cycle control, and apoptosis. Understanding how these genetic modifiers affect ovarian cancer risk may help improve personalized risk prediction, guide prevention and surveillance strategies, and provide new insights into disease mechanisms and potential therapeutic targets.

## 1. Introduction

Among cancers of the female reproductive system, ovarian cancer is the second most diagnosed, following endometrial cancer [1]. Every year, more than 324,000 women worldwide develop ovarian cancer [2]. The median age at diagnosis is 63 years [3]. More than 70% of cases are identified at advanced stage (FIGO III and IV) [4]. In 2022, ovarian cancer caused 206,839 deaths globally [2]. The presence of pathogenic variants in the *BRCA1* and *BRCA2* genes is associated with an increased risk of ovarian cancer [1,5,6,7]. The estimated lifetime risk of developing ovarian cancer is 40% for PVs in *BRCA1* and 11–27% for PVs in *BRCA2* [8,9].

Several specific factors contribute to the overall risk of the cancer. Numerous studies have examined the influence of reproductive and environmental factors (e.g., oral contraceptives, smoking, parity, and obesity) on the degree of cancer penetrance among carriers of PVs in the *BRCA1* and *BRCA2* genes [6]. Furthermore, common genetic variants have been shown to modify this penetrance. As a result, women carrying the same PV may exhibit varying susceptibility to cancer, depending on the presence of additional genetic variants that modulate the phenotypic expression of a given mutation. The impact of individual genetic modifiers on ovarian cancer risk in carriers of *BRCA1/BRCA2* pathogenic variants remains clinically limited, due to a slight increase in risk. However, the cumulative effect of multiple variants means that their co-occurrence can significantly increase the overall risk, potentially to a clinically significant extent [10,11,12,13,14]. This study aims to analyze and summarize current knowledge regarding the genetic factors that influence ovarian cancer risk in women carrying pathogenic variants in the *BRCA1* and *BRCA2* genes. Additionally, it underscores the significance of investigating genetic modifiers to improve risk assessment and personalized prevention strategies.

## 2. Methodology

The literature search was performed in June 2025, included studies published between 1996 and 2025, and was conducted according to the Preferred Reporting Items for Systematic Reviews and Meta-Analyses (PRISMA) [15] by two separate reviewers who worked independently. The search strategy included the following terms: ‘BRCA1 genetic modifiers ovarian cancer’, ‘BRCA2 genetic modifiers ovarian cancer’, ‘BRCA1 modifiers GWAS’, ‘BRCA2 modifiers GWAS’, ‘BRCA1 CIMBA’, and ‘BRCA2 CIMBA’. Searches were conducted in the PubMed database using the Boolean operator ‘AND’ to refine the results. The study was registered with PROSPERO (Identifier CRD420251268005).

**Figure 1:**
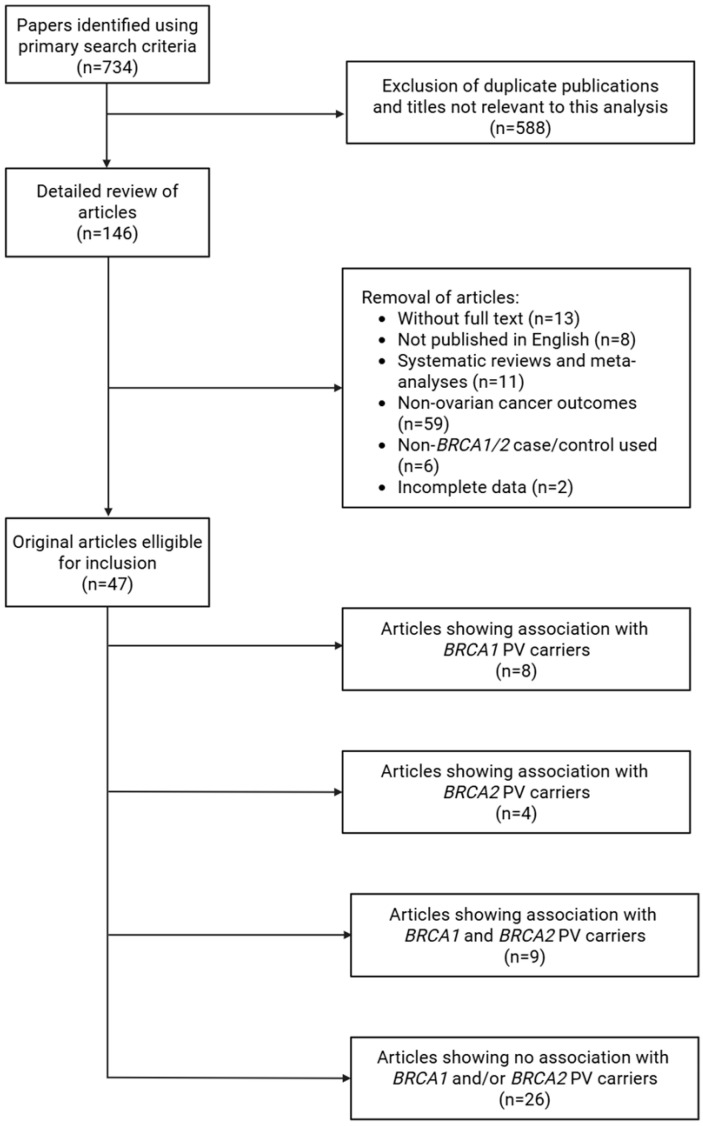
Strategy used to identify the literature about genetic modifiers of ovarian cancer risk.

The detailed results of the literature search strategy are presented in Figure 1. A systematic search was conducted according to defined criteria to identify studies assessing genetic modifiers of ovarian cancer in carriers of PVs in *BRCA1* and *BRCA2.* The initial search retrieved 734 articles. After removing duplicates and studies irrelevant to the research topic, 146 publications remained.

The remaining publications were then reviewed in detail, and papers that failed to meet the inclusion criteria were excluded. Accordingly, articles not available in full text, written in languages other than English, and reviews or meta-analyses were eliminated. Studies unrelated to ovarian cancer outcomes, or those based on non-*BRCA1/BRCA2* cohorts, were also removed. Moreover, papers with incomplete data were not included in the review.

In total, 47 articles met the inclusion criteria and formed the basis of this review. The outcomes from these studies were examined to illustrate the role of genetic modifiers in the development of ovarian cancer among carriers of PVs in *BRCA1* and *BRCA2.* This analysis provides valuable insights into how specific genetic factors may alter ovarian cancer susceptibility within PV carriers.

## 3. Results

### 3.1. Research Approaches to Identify Genetic Modifiers

In recent years, several research strategies have been developed to discover genetic modifiers, ranging from hypothesis-driven candidate gene studies to hypothesis-free genome-wide association studies (GWASs). These strategies vary in scale, extent, and underlying assumptions, and each presents its own set of advantages and limitations.

Initially, the detection of polymorphisms that may modify cancer risk relied on the candidate gene approach. The aim of this method is to conduct research based on hypotheses, focusing on genes that are supposed to play a significant role in disease etiology. Identifying genetic variants associated with cancer involves the comparison of their frequencies between case–control groups—individuals with and without disease [14]. Such studies typically concentrate on functional variants in genes within specific biological pathways, including hormone metabolism and DNA repair [16]. For ovarian cancer, much attention has been given to genes involved in steroid hormone pathways [17,18], DNA repair [19], and regulation of the cell cycle [20,21], as well as known oncogenes [22,23] and tumor suppressors [24,25]. Although the candidate gene studies provide the benefit of more direct biological interpretation of positive results, they have frequently shown limited success. Many initial findings failed to replicate in independent cohorts and the effect sizes of individual variants were too small. Furthermore, another limitation of this approach is its restricted scope, based on a narrow definition of functional variants that focused mainly on coding sequences [16]. Consequently, these challenges restricted the discovery of genetic factors, highlighting the need to develop new, more objective strategies to improve insight into genetic modifiers of cancer risk.

Another alternative approach to the limitations of candidate gene studies is genome-wide association studies (GWASs). GWASs are case–control studies used to identify single nucleotide polymorphisms (SNPs) and copy number variants (CNVs) distributed throughout the genome that influence the risk of diseases such as ovarian cancer [26]. This strategy does not depend on prior assumptions about gene function, allowing for the discovery of entirely novel loci [14]. GWASs are typically conducted using existing resources like biobanks and disease cohorts. The key principles of the analysis are the homogeneity of the groups, the large size of the study and control groups, and the need to validate the results [26]. Most often, genotyping is performed using microarrays and takes place in several stages. The first phase of a GWAS is the “discovery” phase, where SNPs and CNVs potentially associated with the risk of disease are initially identified in a small proportion of samples. Then, the “discovery” phase is followed by a series of “replication studies” to confirm the potential correlation based on subsequent studies on much larger groups of case and control [26,27]. The most strongly associated SNPs and CNVs are chosen as a potential markers that may affect the disease. For GWASs, a very stringent threshold of statistical significance is adopted—*p* < 5.0 × 10^−8^—to minimize the number of false positives [28]. In the context of ovarian cancer in the general population, GWASs identified more than 20 susceptibility variants, including loci at 9p22.2, 8q24.21, 2q31.1, 17q21.31, and 19p13.11 [29,30,31,32,33]. Although these findings have led to a better understanding of the genetic factors that influence ovarian cancer and revealed previously unidentified genomic regions, GWASs remain limited by the lack of prior insight into functional variants or biological pathways involved [16]. Moreover, GWASs explain only a minor fraction of the heritability of most complex traits, as many associated variants have very small effect sizes that fail to reach the stringent genome-wide significance threshold [34,35]. A substantial part of the so-called missing heritability likely stems from undetected common variants of weak effects, rare and ultra-rare variants, as well as gene–gene and gene–environment interactions that are difficult to capture using standard GWAS designs [36]. Additionally, linkage disequilibrium often obscures casual variants, with most signals mapping to non-coding regions whose interpretation remain challenging [37]. Another important limitation is the lack of diversity in study populations. According to the GWAS Diversity Monitor data, as of September 5, 2025, over 87% of GWAS participants are of European ancestry, while only around 5% are of Asian descent, highlighting the strong European bias in current datasets (www.gwasdiversitymonitor.com). Early genome-wide SNP arrays were based on European reference panels, leading to limited coverage in non-European populations. Although newer trans-ethnic arrays have improved diversity, many groups remain underrepresented, and reliance on European-based data, such as on the ExomeChip, continues to restrict GWASs in identifying variants associated with diseases across global populations [38]. Most GWASs have been developed primarily in populations of European ancestry, which limits their predictive accuracy and generalizability in non-European populations. For example, an OncoArray GWAS in an Asian cohort identified both shared and population-specific risk loci compared with European studies, illustrating genetic heterogeneity across ancestries [39]. However, the majority of PRS models for ovarian cancer remain derived from European populations, which may reduce their predictive accuracy and generalizability in non-European populations due to differences in allele frequencies, linkage disequilibrium patterns, and genetic architecture. This lack of diversity in discovery datasets may thus limit effective risk stratification and exacerbate disparities in genetic risk prediction for ovarian cancer across global populations.

A comparison of methodological differences between the candidate gene approach and GWASs is provided in Table 1.

A prominent example of an international effort integrating these approaches to study cancer susceptibility is the Consortium of Investigators of Modifiers of BRCA1/2 (CIMBA), which has been established to identify genes that modify the risk of breast and ovarian cancer among carriers of PVs in the *BRCA1* and *BRCA2* genes. The main purpose of the CIMBA is to ensure adequate sample size to enable large-scale studies to be conducted and the impact of genetic modifiers to be reliably assessed [13]. To uncover genetic loci associated with breast and ovarian risk in PV carriers, the CIMBA has applied four main strategies, including GWASs in samples of carriers, association studies of common variants from the general population, and meta-analyses combining carriers and general population data, as well as fine-scale mapping of identified risk loci [16]. Large-scale studies have identified several SNPs associated with ovarian cancer risk and shown that many of these variants in *BRCA1* and *BRCA2* PV carriers are also consistent with those found in the general population [8,40,41]. This confirms the use of GWAS data from the general population to identify risk modifiers in PV carriers.

**Table 1:** Key differences between candidate gene studies and GWASs.

Feature	Candidate Gene Studies	GWAS
**Prior hypothesis**	Required	None
**Number of variants analyzed**	Limited to tens or hundreds of variants	Genome-scale, millions of SNPs
**Selection of genes**	Yes	No
**Type of variants analyzed**	Can detect rare/non-SNP variants (e.g., VNTRs)	Focus on common SNPs (<1% MAF), some rare variant coverage via arrays or imputation
**Sample size**	Usually small (hundreds)	Large (tens of thousands)
**Objective**	Validation of biologically plausible variants	Discovery of novel susceptibility loci
**Statistical power**	High for tested variants	Lower due to multiple testing corrections
**Limitations**	Limited genomic scope, high selection and publication bias, poor replicability, relies on prior knowledge	Requires large sample sizes, high multiple testing, interpretation challenges
**False positive rate**	Large	Low
**False negative rate**	Low	Large
**Significance threshold**	*p* < 0.05	*p* < 5.0 × 10^−8^
**Common analysis methods**	TaqMan genotyping	Microarrays

Note: Data collected from several articles [28,42,43,44,45,46,47].

### 3.2. BRCA1 Genetic Modifiers

Studies conducted on patients carrying a pathogenic *BRCA1* gene variant have demonstrated the influence of additional genetic factors on the risk of developing ovarian cancer. Consequently, certain polymorphisms may either increase or decrease cancer susceptibility. Research to date has primarily focused on genes considered functionally important, either through direct interaction with *BRCA1* or through their relevance to tumor biology. All genes discussed are presented in Table 2.

Before discussing individual loci, it is important to note that several genetic variants have been identified as increasing the risk of ovarian cancer in *BRCA1* PV carriers. These risk-enhancing polymorphisms act through diverse biological pathways, including DNA repair, metabolism, cell adhesion, and apoptosis regulation.

The *OGG1* gene is involved in base excision repair. SNP rs2304277 was found to be associated with an increased risk of ovarian cancer in *BRCA1* PV carriers (HR = 1.12; 95% CI: 1.03–1.21; *p* = 4.8 × 10^−3^). In a dominant model, which included carriers of at least one pathogenic allele, the effect was stronger (HR = 1.19; 95% CI: 1.08–1.30; *p* = 6 × 10^−4^) [48].

Building on evidence from DNA repair-related variants, metabolic genes have also been implicated as potential modifiers. For the *MTHFR* c.665C>T polymorphism, TT homozygotes demonstrated a significantly increased risk of ovarian cancer. In confounder-adjusted analyses, the risk was OR = 2.09 (95% CI: 0.74–5.82), while after adjusting for consanguinity in the clustered analysis, the risk remained elevated at OR = 1.64 (95% CI: 1.15–2.34). An even stronger effect was observed when the TT genotype was compared with the combined CC + CT genotypes; in this setting, the adjusted OR was 2.7 (95% CI: 1.01–7.21), and after adjusting for familial clustering, 1.94 (95% CI: 1.38–2.74). These findings indicate that in the Polish population, *BRCA1* carriers with the *MTHFR* TT genotype have approximately a two-fold higher risk of ovarian cancer compared with other genotypes [49]. This association was not replicated in another two studies conducted in Italian [50] and multicenter cohorts [51].

Another group of candidate modifier genes includes those encoding proteins involved in cell adhesion and signaling. Integrins play a key role as receptors responsible for cell adhesion and signaling. The functional c.176T>C polymorphism in the integrin ß3 subunit, encoded by *ITGB3*, has been shown to influence various properties of ß3-expressing cells. In a case–control study evaluating *BRCA1* PV carriers in the Polish population, the C allele occurred significantly more frequently in ovarian cancer cases than in controls. Among *BRCA1* PV carriers, the C allele was present in 19% of ovarian cancer cases (57 of 292 alleles) and in 11% of controls (61 of 560; *p* < 0.001). C allele carriers had a significantly higher risk of ovarian cancer than individuals with the TT genotype (OR = 2.08; 95% CI: 1.33–3.27). After accounting for consanguinity in clustered analysis, the association remained significant (OR = 1.97; 95% CI: 1.53–2.53). The median age at diagnosis was comparable in Pro-allele carriers and non-carriers—46 years (25–75) versus 47 years (27–71), respectively (*p* = 0.87)—suggesting that this variant does not significantly affect the age of disease onset in this group [52]. A large multicenter analysis including PV carriers from 34 CIMBA studies did not replicate the results from the Polish population. Only marginal evidence for an increased risk in *BRCA1* PV carriers was found, which disappeared when the Polish cohort was excluded [53]. These findings suggest that the effect of *MTHFR* may be restricted to the Polish population.

As research broadened to include genes involved in apoptotic regulation, additional modifiers were identified. Dysregulation of apoptosis is known to contribute to carcinogenesis; therefore, variants in cell death pathways represent biologically plausible candidates. The *DR4* c.683A>C polymorphism (TRAIL-R1) was significantly associated with an increased risk of ovarian cancer in *BRCA1* PV carriers, with an odds ratio of 1.78 (95% CI: 1.24–2.55; *p* = 0.009; n = 557). These findings suggest that the *DR4* c.683A>C variant may contribute to elevated risk in this population [54].

Further support for genetic modifiers also emerges from studies of ubiquitination-related pathways. The *TRIM61* rs4691139 polymorphism was identified as a significant modifier of ovarian cancer risk in *BRCA1* PV carriers. Carriers of this variant demonstrated an increased risk of disease, with HR = 1.20 (95% CI: 1.17–1.38). Notably, this association was specific to *BRCA1* PV carriers and was not observed in *BRCA2* carriers or the general population [55].

Evidence also points to the role of proto-oncogenes in modifying ovarian cancer risk. The *HRAS1* gene contains a variable number of tandem repeat (VNTR) polymorphisms, which may influence cancer susceptibility through altered expression or regulation. In *BRCA1* PV carriers, the presence of *HRAS1* variants was associated with an approximately 2.11-fold increased risk of ovarian cancer (*p* = 0.015). Importantly, this effect was limited to ovarian cancer; this variant did not significantly increase breast cancer risk in the same cohort [56].

Finally, regulatory polymorphisms in genes influencing *TP53* activity have also been implicated. The *MDM2* SNP309 c.14+309T>G variant was associated with an earlier onset of ovarian cancer in *BRCA1* PV carriers, with TG and GG genotypes conferring a 53% (OR = 1.53; 95% CI: 1.07–2.19; *p* = 0.020) and 92% (OR = 1.92; 95% CI: 1.19–3.10; *p* = 0.009) increase in risk, respectively, compared with the TT genotype. On a molecular level, the G allele increases *MDM2* expression and reduces p53 activity, which may accelerate tumorigenesis in *BRCA1*-deficient cells [57,58]. At the same time, studies have shown that the c.14+285G>C variant reduces the risk of ovarian cancer, and the 285C/309G haplotype may partially counteract the increased risk associated with the 309G allele. Excluding carriers of this haplotype strengthens the association between SNP309G and elevated ovarian cancer risk, confirming the modulatory effect of SNP285 [58].

Several studies have identified polymorphisms associated with a reduced risk of ovarian cancer among *BRCA1* PV carriers. These protective variants frequently affect regulatory elements or apoptosis-related pathways and may mitigate tumor development despite *BRCA1* dysfunction.

There is also evidence that a CNV mapped to *CYP2A7* may influence ovarian cancer risk in *BRCA1* PV carriers. The study showed that the presence of this deletion was associated with a substantially reduced risk of disease, corresponding to a hazard ratio of approximately 0.50 (*p* = 0.007). Functional analyses revealed that the deletion encompasses an enhancer region active in ovarian, but not breast, tissue. This suggests that its protective effect may stem from altered regulation of ovarian-specific genes. The deletion region may interact with the *EGLN2* gene, potentially modulating the expression of genes involved in oxidative stress response and genomic stability. Collectively, these findings indicate that *CYP2A7* deletion may confer protection against ovarian cancer in *BRCA1* PV carriers [59].

Apoptosis-related genes continue to play a central role in understanding protective genetic effects. *CASP8*, a key component of the extrinsic apoptotic pathway, contains the c.863G>C variant, previously linked to reduced breast cancer risk in the general population. In *BRCA1* PV carriers, individuals carrying the C allele had a significantly lower risk of ovarian cancer, with a per-allele HR of 0.69 (95% CI: 0.53–0.89; *p* = 0.008). Heterozygotes had an HR of 0.73 (95% CI: 0.55–0.98), while homozygotes of the rarer allele had an HR of 0.31 (95% CI: 0.10–0.94). These findings suggest that the CASP8 c.863G>C polymorphism may exert a protective effect by enhancing apoptosis in cells with DNA damage [60].

Of the polymorphisms discussed, only one—the SNP located in *TRIM61* (rs4691139)—reached the GWAS significance threshold (*p*-value < 5.0 × 10^−8^).

**Table 2:** Polymorphisms found to be associated with a modified ovarian cancer risk for *BRCA1* pathogenic variant carriers.

Gene	Locus	Polymorphism	Sample Size	Unaffected/ Affected	HR/OR/RR (95% CI)	*p*-Value	Genotyping Platform	Function	Ref
*OGG1*	3p25.3	rs2304277	15,245	12,783/2462	HR: 1.12 (1.03–1.21)	4.8 × 10^−3^	iCOGS	Base excision repair (oxidative DNA damage)	[48]
*MTHFR*	1p36.22	rs1801133	426	2800/146	OR: 2.7 (1.01–7.21)	0.047	PCR-based	Folate metabolism	[49]
*ITGB3*	17q21.32	rs5918	426	280/146	OR: 2.08 (1.33–3.27)	0.001	Taqman	Integrin β3—cell adhesion	[52]
*DR4*	8p21.3	rs17088993	557	480/77	HR: 1.78 (1.24–2.55)	0.009	Taqman	Apoptosis (TRAIL receptor)	[54]
*TRIM61*	4q32.3	rs4691139	14,350	12,070/2280	HR: 1.20 (1.17–1.38)	3.4 × 10^−8^	iCOGS, iPLEX	Protein ubiquitination	[55]
*HRAS1*	11p15.5	VNTR	154	112/42	RR: 2.11 (1.15–4.18)	0.015	VNTR genotyping	Oncogene; altered expression	[56]
*MDM2*	12q15	rs2279744	2626	2465/161	OR: 1.92 (1.19–3.10)	0.009	Taqman	Regulation of p53	[58]
*MDM2*	12q15	rs117039649	2626	2465/161	OR: 1.73 (1.23–2.45)	0.002	Taqman	Modulates promoter activity regulating MDM2 expression	[58]
*CYP2A7*	19q13.2	CNV deletion	2319	1962/357	RR: 0.5 (0.2–1.27)	0.007	Illumina Human610-Quad	Enhancer deletion reducing ovarian-specific risk	[59]
*CASP8*	2q33.1	rs1045485	4844	4268/576	HR: 0.69 (0.53–0.89)	0.008	Taqman	Initiation of apoptosis	[60]

### 3.3. BRCA2 Genetic Modifiers

The risk of developing ovarian cancer in individuals carrying pathogenic variants of the *BRCA2* gene variants may be influenced not only by the presence of the mutation itself but also by the influence of additional genetic modifiers. Research indicates that specific variants in selected genes may modify the penetrance of ovarian cancer in *BRCA2* PV carriers. Much of the existing work focuses on polymorphisms (SNPs) located in genes involved in key cellular processes, particularly DNA repair pathways. All genes discussed below are summarized in Table 3.

Similarly to BRCA1, several genetic variants have been identified that may increase the susceptibility to ovarian cancer among carriers of pathogenic BRCA2 mutations. These modifiers are primarily located in genes related to DNA repair pathways, potentially influencing genomic stability and cancer development.

Haplotypes in DNA repair genes were found to modify the risk of ovarian cancer among *BRCA2* PV carriers, as confirmed by the findings of Rebbeck et al. In the *ATM* gene, the haplotype “TTGGC,” with a frequency of 0.9%, increased ovarian cancer risk with an HR of 10.93 (95% CI: 4.43–26.96) compared to the reference haplotype. It differs from the reference haplotype at SNPs rs664982 and rs664143. Moreover, in *BRIP1*, haplotypes “B” (frequency 2.0%) and “G” (frequency 2.4%) also increased risk, with HRs of 6.59 (95% CI: 1.10–39.65) and 7.28 (95% CI: 1.67–31.82), respectively. Haplotype “B” differs at SNP rs4988340, and haplotype “G” differs at SNPs rs12453935, rs169456280, and rs10515211. Furthermore, in *BARD1*, the rare haplotype “TTTCGGCT” elevated ovarian cancer risk to HR = 4.62 (95% CI: 1.31–16.31) and differed from the reference haplotype at SNP rs6712055. In *MRE11*, haplotype “C”, with a frequency of 18.4%, increased risk with HR = 2.33 (95% CI: 1.39–3.91), while a very rare haplotype raised the HR to 5.13 (95% CI: 1.24–21.24). Similarly, haplotype “C” in *RAD51* (frequency 11.9%) also increased ovarian cancer risk, with HR = 3.53 (95% CI: 1.77–7.05) [61].

In addition to variants associated with increased risk, several genetic changes have also been identified that may reduce the likelihood of developing ovarian cancer in *BRCA2* pathogenic variant carriers. These protective modifiers often influence mechanisms of DNA repair, cellular stress responses, or telomere stability, collectively contributing to a decreased penetrance of the disease. Their identification highlights the complexity of genetic interactions shaping cancer risk in this group of patients.

SNP rs1801320 (135G>C) in the *RAD51* gene showed a tendency toward reduced ovarian cancer risk (HR = 0.40; 95% CI: 0.05–3.40), although the small number of variant carriers limited statistical power.

The rs34259 polymorphism in the *UNG* gene has also been shown to be associated with a reduced risk of ovarian cancer in carriers of a pathogenic variant in the *BRCA2* gene. Individuals carrying the rs34259 allele exhibited significantly reduced UNG mRNA levels and lower UNG1 protein expression relative to non-carriers. Additionally, cells with this allele showed decreased telomeric DNA damage, including reduced 8-oxoguanine levels and diminished uracil accumulation. The rs34259 allele was also associated with decreased susceptibility to oxidative stress and reduced telomeric uracil levels specifically in *BRCA2* PV carriers. Women harboring this variant had shorter age-adjusted telomeres (*p* < 0.05) and a trend toward a higher proportion of very-short telomeres (<3 kb) [12,48,62].

Two variants in the *TDG* gene (rs167715 and rs4135087) were associated with ovarian cancer risk reduction in trend analyses (*p* < 0.01). Hazard ratio estimates suggested a protective effect, although wide confidence intervals reflected the limited sample size of the *BRCA2* subgroup. Moreover, for the *PARP2* gene, variant rs3093926 showed a similarly strong association (*p*-trend < 0.01). Imputation for the linked SNP rs61995542 suggested reduced risk (HR ≈ 0.67). However, the authors emphasized that results for *TDG* and *PARP2* require further validation due to the risk of false positives associated with insufficient statistical power [48].

Finally, evidence suggests that a locus on chromosome 9p22.2 may reduce ovarian cancer risk in *BRCA2* PV carriers. The strongest associated SNP, rs62543585 (MAF~0.20), was linked to a decreased risk (HR per allele = 0.69; 95% CI: 0.59–0.80; *p* = 1.0 × 10^−6^). All potentially causal variants are located within ~45 kb upstream of the BNC2 gene in a regulatory region, which may influence its expression [63].

Of the polymorphisms discussed, none reached the GWAS significance threshold (*p*-value < 5.0 × 10^−8^).

**Table 3:** Polymorphisms found to be associated with a modified ovarian cancer risk for *BRCA2* pathogenic variant carriers.

Gene	Locus	Polymorphism	Sample Size	Unaffected/ Affected	HR (95% CI)	*p*-Value	Genotyping Platform	Function	Ref
*ATM*	11q22.3	Haplotype (rare)	856	809/47	10.93 (4.43–26.96)	0.022	SNPlex	DNA damage response	[61]
*BRIP1*	17q22	Haplotype B	856	809/47	6.59 (1.10–39.65)	0.001	SNPlex	*BRCA1*-interacting helicase	[61]
*BRIP1*	17q22	Haplotype G	856	809/47	7.28 (1.67–31.82)	0.001	SNPlex	*BRCA1*-interacting helicase	[61]
*BARD1*	2q34	Haplotype (rare)	856	809/47	4.62 (1.31–16.31)	0.003	SNPlex	*BRCA1*-binding tumor suppressor	[61]
*MRE11*	11q21	Haplotype C	856	809/47	2.33 (1.39–3.91)	0.003	SNPlex	DNA break repair in the MRN complex	[61]
*MRE11*	11q21	Haplotype (rare)	856	809/47	5.13 (1.24–21.24)	0.003	SNPlex	DNA break repair in the MRN complex	[61]
*RAD51*	15q15.1	Haplotype C	856	809/47	3.53 (1.77–7.05)	0.01	SNPlex	Homologous recombination	[61]
*UNG*	12q23.2	rs34259	8211	7580/631	0.80 (0.69–0.94)	7.6 × 10^−3^	iCOGS	Base excision repair (uracil DNA glycosylase)	[48]
*TDG*	12q24.1	rs4135087	8211	7580/631	1.32 (1.09–1.59)	2.8 × 10^−3^	iCOGS	Base excision repair	[48]
*TDG*	12q24.1	rs167715	8208	7577/631	0.76 (0.62–0.94)	7.4 × 10^−3^	iCOGS	Base excision repair	[48]
*PARP2*	14q11.2	rs3093926	8211	7580/631	0.64 (0.49–0.84)	1.5 × 10^−3^	iCOGS	DNA repair (BER)	[48]
upstream of *BNC2*	9p22.2	rs62543585	5837	5314/523	0.69 (0.59–0.80)	1 × 10^−6^	iPLEX	Regulatory region affecting *BNC2*	[63]

### 3.4. BRCA1 and BRCA2 Genetic Modifiers

Several studies have shown that specific variants, particularly SNPs, may affect the risk of ovarian cancer in carriers of PVs in *BRCA1* as well as those with PVs in *BRCA2.* The results confirm that certain polymorphisms are significantly associated with modified ovarian cancer risk, either by increasing or decreasing cancer susceptibility, with similar effects observed in individuals carrying pathogenic variants in *BRCA1* or *BRCA2* [8,40,41,55,64,65,66,67].

Among the eleven identified variants, eight have been associated with an increased risk of developing ovarian cancer. Ding et al. showed that a polymorphism in *IRS1* (rs1801278), which influences insulin signaling pathways and the insulin-like growth factor, acts as a modifier of ovarian cancer risk in *BRCA1* and *BRCA2* PV carriers. Their findings reported that the presence of this variant was associated with an approximately 1.5-fold increased risk in *BRCA1* PV carriers and more than a two-fold increase in *BRCA2* PV carriers [65]. In another study, two variants (rs58722170 and rs17329882) were found to be significantly associated with higher epithelial ovarian cancer (EOC) risk in *BRCA* PV carriers [41]. The strongest correlation with ovarian cancer showed the polymorphism located in *RSPO1* (rs58722170), which encodes the R-spondin-1 involved in cell proliferation and cancer development [41,68]. There is also evidence that patients with PVs in both *PPARGC1A*, a gene-coding coactivator of PPARγ, and *BRCA1/2* developed ovarian cancer earlier than carriers of PVs in only one of the analyzed genes. Moreover, most of PVs in *PPARGC1A* detected by whole-genome sequencing (WGS) in a study conducted by Zhu et al. were located in non-coding regions, emphasizing the need to explore regulatory regions in the search for genetic modifiers of cancer susceptibility [67]. An association with a higher risk of developing ovarian cancer in *BRCA* PV carriers has also been demonstrated for the polymorphisms in *BABAM1* (rs8170), *MRPL34* (rs67397200), *PLEKHM1* (rs17631303), and *TIPARP* (rs2665390) (Table 4).

There is likewise evidence of polymorphisms that may decrease the risk of developing ovarian cancer in *BRCA* PV carriers. Yarmolinksy et al. recently reported that genetically proxied inhibition of HMG-CoA reductase—analogous to the pharmacological effect of statin therapy—was associated with an almost two-fold reduced risk of ovarian cancer in carriers of PVs in *BRCA1/2* (HR = 0.69, *p* = 0.01). Remarkably, this protective effect was specific to polymorphisms in *HMGCR* and was not observed for SNPs in other lipid-related genes, such as *NPC1L1* or *PCSK9*, nor with genetically predicted reductions in circulating LDL cholesterol levels. This suggest that the mechanism of risk reduction may involve a specific role of the mevalonate pathway, regulated by the activity of HMG-CoA reductase, in the biology of ovarian epithelial cells [64]. Additionally, the “C” allele of the SNP rs3814113 in *BNC2* has been found to be associated with a reduced risk of ovarian cancer in *BRCA* PV carriers. For instance, individuals carrying PVs in *BRCA1* with the “CC” genotype were predicted to have a 33% risk of developing ovarian cancer risk by the age of 80 [40]. The polymorphism in *LINC00824* (rs10088218), which is located more than 700 kb downstream (3′) of *MYC*, may also decrease the risk, and its suggested function involves a distant regulation of *MYC* expression [8].

Of the polymorphisms discussed, only one—the SNP located in *BNC2* (rs3814113)—reached the GWAS significance threshold (*p*-value < 5.0 × 10^−8^).

### 3.5. Candidate BRCA1/BRCA2 Genetic Modifiers with Unconfirmed Effect

Although numerous studies have identified polymorphisms that may modify ovarian cancer risk among carriers of PVs in *BRCA1* and/or *BRCA2*, not all reported associations were statistically significant. Several investigations evaluating candidate variants or loci, selected either on the basis of biological relevance or genome-wide studies, have failed to demonstrate significant association with ovarian cancer risk among *BRCA1/2* PV carriers.

Among these studies, variants located within the *BRCA1* and *BRCA2* genes themselves were examined as potential modifiers of cancer risk in carriers of PVs. In *BRCA1*, the intronic rs5820483 SNP, which affects exon 11 alternative splicing and has been linked to breast cancer risk, showed no significant association with ovarian cancer susceptibility [69]. Additionally, the nonconservative coding variant N372H in *BRCA2* was not associated with ovarian cancer risk in *BRCA1* PV carriers, whereas only a borderline effect was reported for the 5′ untranslated region variant 203G>A [70].

Polymorphisms in other DNA repair- and cell cycle-related genes were also extensively evaluated. In *TP53*, the 16 bp duplication in intron 3 (c.97_147ins16bp) and the p.Arg72Pro (c.215C>G), as well as variants c.441G>C and c.1798G>A, showed no association with ovarian cancer risk in *BRCA1* and/or *BRCA2* PV carriers, although initial signals in unselected populations were reported. Many analyses were limited by small numbers of ovarian cancer cases and a lack of ovarian-specific data, with associations often reflecting combined breast and ovarian cancer outcomes [71,72,73,74]. Common variants in *RAD51L1* (rs999737 and rs10483813), which encodes the DNA repair protein RAD51 homolog 2, also showed no effect on ovarian cancer risk [75]. Likewise, a comprehensive evaluation of polymorphisms in *ERCC2*, *XRCC1*, *XRCC2*, *XRCC3,* and *LIG4* revealed no significant associations with BRCA1-associated ovarian cancer risk in carriers of common Polish founder pathogenic variants [71].

Hormone-related candidate modifiers were also extensively investigated. Studies exploring CAG and GGC repeat lengths in exon 1 of the *AR* gene consistently reported no association with ovarian cancer risk, although some effects on age at diagnosis or survival were suggested [76,77,78,79,80]. Similarly, the *PROGINS* allele of the progesterone receptor (*PGR*) was not linked to ovarian cancer risk overall, with subgroup-specific effects observed only in relation to OCP use [81]. Additional *PGR* variants, including c.331G>A and 88022ins306, also showed no significant association [71]. Polymorphisms in the estrogen receptor 1 (*ESR1*) gene (rs2046210 and rs9397435) were likewise investigated and did not significantly influence ovarian cancer risk [75].

Results from a multicenter study also indicated that the *PHB* c.1630C>T polymorphism was not linked to either breast or ovarian cancer when each cancer type was considered individually. However, when breast and ovarian cancer were analyzed together, *BRCA1* carriers homozygous for the rare *PHB* 1630TT genotype exhibited a modestly increased risk of both cancers, although no effect was detected under a multiplicative model [51]. Likewise, the same polymorphisms of *PHB* were not associated with ovarian cancer risk in Polish *BRCA1* PV carriers [82].

The N314D variant of the *GALT* gene, implicated in galactose metabolism, was not associated with EOC risk, also in analyses stratified by *BRCA1* and *BRCA2* PV status, and no modifying effect was observed across histological subtypes or clinical subgroups [83]. Other metabolic-related genes, including polymorphisms in *TYMS*, *COMT*, CYP11A1, CYP17A1, CYP19A1, and HSD17, were also analyzed, but none showed a significant effect on ovarian cancer susceptibility in carriers of *BRCA1* PV [71].

The *KRAS* 3′UTR rs61764370 SNP was evaluated in two independent studies, neither of which demonstrated an association with ovarian cancer risk in *BRCA1* and *BRCA2* PV carriers. In the first study conducted by Pharoah et al., no relationship was observed between the rs61764370 genotypes and risk of EOC among *BRCA1* PV carriers [84]. Consistently, a larger study confirmed the absence of an association with ovarian cancer risk in both *BRCA1* and *BRCA2* PV carriers [85].

Variants in *Klotho* (KL-VS, rs9536314) and *TERT* were likewise investigated for their potential role in modifying ovarian cancer risk. The KL-VS polymorphism showed no significant effect either for *BRCA1* or *BRCA2* PV carriers [86]. Analyses of telomere length, a proxy of *TERT* activity, revealed that PV carriers, particularly in *BRCA2* families, had longer telomeres than their non-carrier relatives, but this did not correspond to an increased risk of ovarian cancer [87].

Research on other candidate variants has largely failed to demonstrate a clear modifying effect on ovarian cancer risk in *BRCA1/2* PV carriers. At chromosome 2q24.2, a significant loss at the D2S156 marker was observed in sporadic ovarian cancer, but no such alteration was found in the *BRCA1*-associated group [88]. Similarly, several SNPs were previously suggested to influence ovarian cancer risk, including rs12025623 and rs1767429 in *ALP* among *BRCA1* PV carriers, and rs2233025 in *MAD2L2* among BRCA2 PV carriers [89]. Additional analyses of *AIB1*, *HER2*, *IL6*, *LRP1*, *TGFB*, *TGFBR1, NOTCH2*, *FGF13*, and SNPs at 6p24 (rs9348512) and 2p22 (rs184577) also failed to demonstrate any modifying effect on susceptibility to ovarian cancer [90]. Detailed results are summarized in Supplementary Files Table S1.

Overall, these findings illustrate the difficulty of identifying genetic modifiers of ovarian cancer risk in *BRCA1* and *BRCA2* PVs. The predominance of negative findings may reflect modest effects of individual variants, limited study size, or interactions between genes and genes and the environment. This underscores the need for large, well-designed studies with comprehensive data to identify modifiers of ovarian cancer risk [91].

### 3.6. Genetic Modifiers and Polygenic Risk Models

Investigating genetic modifiers and additional risk-modulating factors reveals that the susceptibility of developing ovarian cancer is not constant and varies among carriers of PVs in *BRCA1* and *BRCA2.* Current clinical preventive strategies for *BRCA1/2* PV carriers include intensive surveillance, chemoprevention, and surgery, such as risk-reducing salpingo-oophorectomy (RRSO), which significantly decreases cancer risk but may also have long-term consequences [92,93,94]. Incorporating risk-modifying factors into risk prediction models could refine current estimations of cancer susceptibility. This may enable the development of more personalized preventive and therapeutic strategies, optimized for an individual risk profile and differentiating individuals with a higher or lower risk of developing cancer [95].

Although each individual polymorphism described confers only a small increase in disease risk, the combined effect of multiples variants, summarized as a polygenic risk score (PRS), may result in a substantially higher overall risk estimate [55]. Some studies have assessed the effect of the PRS on ovarian cancer risk, using variants linked to EOC risk primarily at genome-wide significance in PV carriers and the general population [95,96,97,98].

Several of the identified genetic modifiers have already been incorporated into current ovarian cancer risk prediction models. Of the SNPs highlighted in our review, seven variants (*WNT4* rs56318008, *RSPO1* rs58722170, *SYNPO2* rs17329882, *TRIM61* rs4691139, *GPX6* rs116133110, *BNC2* rs3814113, and *ABO* rs635634) have been integrated in PRSs and tested for ovarian cancer risk in *BRCA1* and *BRCA2* PV carriers [95,96]. However, it should be noted that the inclusion of a variant in a polygenic risk score does not necessarily imply strong or replicated evidence; variants are incorporated based on their potential contribution to cumulative risk even if individual effects are modest, and clinical utility remains limited in many settings [99]. In a study conducted by Kuchenbaecker et al., a population-based PRS, including 17 SNPs, demonstrated a statistically significant association with ovarian cancer risk in both *BRCA1* and *BRCA2* PV carriers. The magnitude of this association was significantly larger in individuals with *BRCA2* PVs (HR = 1.49, 95% CI [1.34–1.65]), compared to *BRCA1* PV carriers (HR = 1.28, 95% CI [1.22–1.34]). Importantly, this analysis also identified a significant correlation between age and PRS in *BRCA1* PV carriers (*p* = 0.003), suggesting that the genetic risk of ovarian cancer conferred by the PRS may be stronger in younger individuals [95]. In another study, Barnes et al. developed two PRS models: a 30-SNP score for overall EOC risk (PRS_EOC_) and a 22-SNP score based on variants specifically associated with high-grade serous EOC (PRS_HGS_). Both models were strongly associated with EOC risk among *BRCA1* (PRS_EOC_: HR = 1.31, 95% CI [1.24–1.39]; PRS_HGS_: HR = 1.32, 95% CI [1.25–1.40]) and *BRCA2* (PRS_EOC_: HR = 1.43, 95% CI [1.29–1.59]; PRS_HGS_ = 1.44, 95% CI [1.30–1.60] PV carriers and showed similar correlations. These associations did not differ significantly by age or country of origin. Additionally, for *BRCA1* PV carriers, the strength of correlation varied by the location of the pathogenic variant, with higher risk estimates for variants in the central region of *BRCA1* (central region HR = 1.50, 95% CI [1.35–1.66]; 5′ to c.2281 region HR = 1.30, 95% CI [1.18–1.42]; c.4072 to 3′ region HR = 1.21, 95% CI [1.10–1.33]) [96]. A S4 PRS model developed by Dareng et al., based on 27,240 SNPs, similarly showed a significant link with ovarian cancer in *BRCA1* (HR = 1.36, 95% CI [1.29–1.43]) and *BRCA2* (HR = 1.49, 95% CI [1.35–1.64]) PV carriers. This model demonstrated moderate discriminatory performance (AUC: 0.59 for *BRCA1*; 0.62 for *BRCA2*), supporting its potential role in individual risk stratification [97]. Collectively, these findings demonstrate that polygenic risk scores capture meaningful inter-individual variability in cancer susceptibility among *BRCA1* and *BRCA2* PV carriers. This provides a quantitative framework for risk stratification, forming the basis for subsequent evaluation of the clinical applicability of PRS-based approaches.

### 3.7. Implications and Utility in Clinical Practice

Building on these genetic risk stratification frameworks, recent efforts have focused on translating the PRS into clinical risk prediction models and preventive decision-making. Individual genetic modifiers generally have only modest effects and are unlikely to be clinically useful on their own. In contrast, polygenic risk scores (PRSs), which combine multiple variants, may provide more meaningful risk estimates for future clinical applications.

The findings discussed in this review clearly indicate that PRSs developed using population-based cohorts are also applicable to *BRCA1* and *BRCA2* PV carriers. When taken together with family history and reproductive and hormonal factors that may also modify cancer susceptibility, PRSs can contribute to more refined stratification of EOC risk. Several clinical risk models, such as BOADICEA implemented in the CanRisk tool (www.canrisk.org), have already integrated polygenic components along with high-penetrance variants to improve risk prediction [100,101]. These tools provide reliable data on the risk of EOC and are increasingly applied in clinical practice to support decision-making about preventive strategies, including oral contraceptives, risk-reducing surgeries, and increased surveillance with CA-125 [102]. Moreover, a recent study suggests that integrating the PRS with transvaginal ultrasound (TVU) results, may also enhance the predictive value of screening strategies. Individuals in the highest PRS quartile not only showed a significantly increased risk of developing EOC (OR = 1.77, 95% CI [1.04–3.08]), but also had higher odds of abnormal TVU findings (OR = 1.46, 95% CI [1.17–1.83]), indicating the potential of combined approaches to improve early detection of cancer [103].

Despite current evidence supporting the potential role of PRSs in refining ovarian cancer risk estimates, their clinical application remains limited. There are several challenges that need to be overcome before PRSs can be incorporated into clinical practice. Importantly, the presence of gene–environment interactions suggests that the effect of the PRS on disease risk may differ between individuals depending on environmental factors [104,105]. Moreover, most existing models are based on GWAS data from the European population, which often limits their predictive accuracy in non-European ancestries [106]. To determine the utility of risk prediction, PRS-based screening programs require validation in large, prospective, and randomized clinical trials. Ongoing investigations, including PROVE in Europe (ClinicalTrials.gov identifier: NCT06935344) and PROMISE in the USA (ClinicalTrials.gov identifier: NCT06436248), are focused on determining how PRSs can improve ovarian cancer screening strategies. Beyond clinical effectiveness, the implementation of PRS-based programs also involves addressing a range of social, ethical, and psychological aspects. Key considerations include the acceptance of genetic risk-stratification programs, training of healthcare providers, and analysis of the long-term efficiency of alternative prevention models [107]. Overcoming these challenges, along with the results of ongoing clinical trials, may provide insight into whether PRSs can improve the personalization of ovarian cancer screening programs.

## 4. Discussion

This review summarized the current data on genetic modifiers of ovarian cancer risk in carriers of PVs in *BRCA1* and *BRCA2*. Even though pathogenic variants in these genes remain the strongest risk factors and predictors of hereditary ovarian cancer, expanding evidence indicates that additional genetic factors contribute to variability in risk among carriers. GWASs have been essential in identifying EOC susceptibility variants in the general population, and findings from studies conducted by the CIMBA suggest that some of these loci also act as modifiers of cancer risk among *BRCA1* and *BRCA2* PV carriers. Ongoing research supports the presence of 10 variants to influence ovarian cancer risk in *BRCA1* PV carriers [47,48,49,50,51,52,53,54,55,56], 13 in *BRCA2* PV carriers (including haplotypes) [12,47,57,58,59], and 11 in both groups [8,40,41,55,64,65,66,67,68]. Only two of the discussed SNPs reached the GWAS significance threshold [39,51].

The identification of genetic factors that affect ovarian cancer risk provides an opportunity for more accurate and personalized risk assessment. This makes it possible to differentiate women at significantly higher risk from women at relatively lower risk of developing cancer, and, consequently, to move away from a “one-size-fits-all” approach [108]. More precise risk prediction allows for more effective planning of screening and surveillance, minimizing overtreatment and avoiding unnecessary interventions in women at lower risk, while ensuring that those at higher risk receive more intensive monitoring [95]. A refined risk assessment may also lead to the development of personalized preventive strategies, such as chemoprevention or lifestyle modification. Furthermore, it could influence patients’ decisions regarding interventions such as RRSO, which, although effective in lowering EOC risk, carries significant long-term consequences [92,93,94]. To determine the impact of genetic risk modifiers on ovarian cancer susceptibility, they are incorporated into PRS models, which allow their combined impact on overall risk to be calculated. Seven variants highlighted in our review have been already integrated into PRS models, and their inclusion has shown a significant association with ovarian cancer risk [95,96,97]. Nevertheless, despite these promising findings, prospective and randomized studies are still required to validate these associations and to support their implementation in clinical practice. Two clinical trials are currently underway—PROVE in Europe and PROMISE in the USA—with the aim of providing such validation.

Beyond risk assessment models, an important advantage of investigating genetic modifiers in carriers of *BRCA1* and *BRCA2* PVs is the possibility of developing new therapeutic options. Determining the impact of these variants on ovarian cancer risk may uncover molecular pathways that could be exploited for targeted therapy, potentially improving clinical outcomes for PV carriers. Moreover, research on genetic modifiers provides valuable insight into biological processes underlying ovarian cancer and helps to explain the mechanisms that influence tumor development in individuals with PVs in *BRCA1* and *BRCA2* [109]. Knowledge of these modifiers could also meaningfully influence psychosocial and reproductive decision-making, as more precise risk estimates help carriers reduce uncertainty and distress [110,111]. Finally, research in this area has also contributed to the development of methodological approaches in the design and application of studies, which may translate into studies on modifiers of other hereditary cancer syndromes, as well as other diseases [109].

Despite promising progress in identifying genetic factors that may influence the risk of developing ovarian cancer, significant challenges remain. One of the difficulties is related to the size of the study cohorts. Even though ovarian cancer is the second most common gynecologic malignancy, recruiting large, well-characterized cohorts of affected *BRCA1* and *BRCA2* PV carriers is demanding due to the combination of advanced stage detection and the fact that the only a subset of individuals carry these high-risk pathogenic variants [4]. Because most genetic modifiers display only a modest effect on cancer risk, detecting their influence requires very large datasets. At the same time, PVs in *BRCA1* and *BRCA2* account for only 10–18% of invasive ovarian carcinomas, which further reduces the statistical power of research and makes it difficult to translate the obtained results into clinical practice [5,112]. Beyond this limitation, another difficulty arises from the fact that the current understanding of genetic risk modifiers is mainly based on studies conducted in populations of European ancestry [113]. Since significant variability is observed across populations, the lack of diversity in current studies limits the ability to generalize results to women of different ancestries and raises the possibility that variants identified in one population may not present the same effect in another [106,113]. Overall, restricting the analysis to European cohorts limits the relevance of findings and highlights the necessity of extending studies to other populations.

Ovarian cancer susceptibility is contributed by a complex interaction of factors, where numerous small-effect genetic modifiers act together with environmental influences. This makes it challenging to determine the specific impact of a given factor on overall risk [10]. Furthermore, accurate risk prediction is complicated by the existence of gene–gene interactions, as the effect of a single variant can be modified by the presence or absence of others. There is evidence that genes involved in DNA repair interact with *BRCA1* and/or *BRCA2* and may influence cancer risk in the presence of PVs in *BRCA1/2*. In the study conducted by Rebbeck et al., it was observed that for *BRCA1* PV carriers, genes such as *BRIP1*, *MRE11*, *RAD50,* and *NBS1*, which are integral to homologous recombination and DNA repair pathways, can modulate ovarian cancer risk. Similarly, for *BRCA2* PV carriers, genes including *ATM*, *BRIP1*, *BARD1*, *MRE11*, and *RAD51* may alter ovarian cancer susceptibility [61]. In addition to gene–gene interactions, ovarian cancer risk is also influenced by gene–environment interactions, through which the effect of genetic modifiers may depend on environmental or lifestyle factors. For instance, a comprehensive analysis carried out by Kim et al. showed that the protective effect of oral contraceptives (OCPs) and menopausal estrogen use may vary depending on the genotype of a given gene variant. The SNP rs11658063 in *HNF1B* appeared to modify the effect of menopausal estrogen therapy, as individuals with the GG genotype showed a higher risk of developing ovarian cancer when using hormone therapy (AR = 1.96%, 95% CI [1.59–2.33%]) compared to never users (AR = 1.33%, 95% CI [1.26–1.40%]). Likewise, SNP rs13255292 in *PVT1* altered the protective impact of OCPs, with the strongest ovarian cancer risk reduction in women carrying the TT genotype (OR = 0.53, 95% CI [0.46–0.60]) compared to CC carriers (OR = 0.71, 95% CI [0.66–0.77]) [114]. Understanding these interactions of genetic and environmental factors is crucial for advancing knowledge about the genetic architecture of disease and for accurate clinical risk assessments [115].

Another major challenge is that, despite the progress made through GWASs in identifying genetic modifiers of cancer risk, insight into their biological function in cancer development remains limited. Furthermore, most disease-associated loci identified in GWASs are located in non-coding regions of the genome, and it remains unclear which gene expressions they regulate and in which cell types this occurs [116]. Without knowledge of the functions of identified variants, it is difficult to apply these findings in clinical practice, limiting their inclusion in PRSs or the development of targeted treatment strategies. Developing targeted therapies based on genetic modifiers is further hindered by the extensive heterogeneity of ovarian tumors [117]. Differences between tumors in individual patients, as well as the diversity of cells within a single tumor, result in distinct genetic profiles, which can cause variability in treatment outcomes and, consequently, limit the effectiveness of therapy [118,119].

## 5. Conclusions

In summary, although the identification of genetic modifiers has improved current knowledge of ovarian cancer risk, applying these findings into clinical practice remains challenging. High baseline cancer risk in *BRCA1/2* carriers, small study cohorts, limited knowledge of variant functions, and the complexity interactions between genes and environmental factors, together with tumor heterogeneity, all limit the clinical utility of PRSs. Further research is needed to confirm these approaches and develop personalized prevention and treatment strategies for individuals carrying PVs in *BRCA1* and *BRCA2*. Moreover, ethical consideration and effective patient communication will also be crucial to ensuring the successful implementation of these strategies.

While the identification of genetic modifiers has improved our understanding of ovarian cancer risk, their translation into clinical practice remains challenging. As these limitations are unlikely to be resolved in the near future, the main value of studying such modifiers may lie in uncovering the biological mechanisms underlying cancer susceptibility, rather than in immediate clinical application.

## Figures and Tables

**Table 4:** SNPs found to be associated with a modified ovarian cancer risk for *BRCA* pathogenic variant carriers.

			*BRCA1* (+)				*BRCA2* (+)						
Gene	Locus	SNP	Sample Size	Unaffected/ Affected	HR (95% CI)	***p***-Value	Sample Size	Unaffected/ Affected	HR (95% CI)	***p***-Value	Genotyping Platform	Function	Ref
*IRS1*	2q36.3	rs1801278	1928	1533/395	1.43 (1.06–1.92)	0.019	1064	944/120	2.21 (1.39–3.52)	0.0008	Taqman, iPLEX	Insulin/IGF-1 signaling mediating	[65]
*RSPO1*	1p34.3	rs58722170	15,252	12,790/2462	1.14 (1.05–1.23)	1.5 × 10^−3^	8211	7580/631	1.35 (1.17–1.57)	5.2 × 10^−5^	iPLEX	Wnt/β-catenin signaling	[41]
*SYNPO2*	4q26	rs17329882	15,252	12,790/2462	1.08 (1.00–1.17)	0.042	8211	7580/631	1.15 (1.00–1.13)	0.06	iPLEX	Cell motility and growth	[41]
*BABAM1*	19p13.1	rs8170	5779	4380/1399	1.15 (1.03–1.29)	0.015	3095	2667/428	1.34 (1.12–1.62)	1.9 × 10^−3^	iPLEX	DNA repair-dependent chromatin remodeling	[66]
*MRPL34*	19p13.1	rs67397200	5011	3699/1312	1.16 (1.05–1.29)	3.8 × 10^−4^	3129	2700/429	1.30 (1.10–1.52)	1.8 × 10^−3^	iPLEX	Mitochondrial translation	[66]
*PLEKHM1*	17q21	rs17631303	11,624	9793/1831	1.27 (1.16–1.40)	3.04 × 10^−7^	8107	7481/626	1.32 (1.15–1.52)	1.98 × 10^−4^	iCOGS	Bone resorption	[55]
*TIPARP*	3q25	rs2665390	11,584	9630/1954	1.25 (1.10–1.42)	6.1 × 10^−4^	6697	6126/571	1.48 (1.21–1.83)	1.8 × 10^−4^	iPLEX	Mono-ADP-ribosylation of proteins	[8]
*BNC2*	9p22.2	rs3814113	10,029	8142/1887	0.78 (0.72–0.85)	4.8 × 10^−9^	5837	5314/523	0.78 (0.67–0.90)	0.00055	iPLEX	Skin color saturation	[40]
*LINC00824*	8q24	rs10088218	12,541	10,479/2062	0.89 (0.81–0.99)	0.029	7129	6515/614	0.81 (0.67–0.98)	0.033	iPLEX	IcnRNA class, may regulate gene expression	[8]

## Data Availability

All relevant data are included in the manuscript.

## Supplementary Materials

The following supporting information can be downloaded at: https://www.mdpi.com/article/10.3390/cancers18030354/s1, Table S1: Polymorphisms not significantly associated with ovarian cancer risk in *BRCA1/BRCA2* PV carriers.

## Abbreviations

GWAS	Genome-wide association study
PV	Pathogenic variant
SNP	Single nucleotide polymorphism
CNV	Copy number variant
CIMBA	Consortium of Investigators of Modifiers of BRCA1/2
EOC	Epithelial ovarian cancer
WGS	Whole-genome sequencing
RRSO	Risk-reducing salpingo-oophorectomy
PRS	Polygenic risk score
AUC	Area under the curve
CI	Confidence interval
TVU	Transvaginal ultrasound
OCP	Oral contraceptive
VNTR	Variable number of tandem repeat

## References

[1] Smolarz B., Biernacka K., Łukasiewicz H., Samulak D., Piekarska E., Romanowicz H., Makowska M. (2025). Ovarian Cancer—Epidemiology, Classification, Pathogenesis, Treatment, and Estrogen Receptors’ Molecular Backgrounds. Int. J. Mol. Sci..

[2] Bray F., Laversanne M., Sung H., Ferlay J., Siegel R.L., Soerjomataram I., Jemal A. (2024). Global Cancer Statistics 2022: GLOBOCAN Estimates of Incidence and Mortality Worldwide for 36 Cancers in 185 Countries. CA Cancer J. Clin..

[3] Siegel R.L., Miller K.D., Jemal A. (2020). Cancer Statistics, 2020. CA Cancer J. Clin..

[4] Kehoe S. (2020). FIGO Staging in Ovarian Carcinoma and Histological Subtypes. J. Gynecol. Oncol..

[5] Zhang S., Royer R., Li S., McLaughlin J.R., Rosen B., Risch H.A., Fan I., Bradley L., Shaw P.A., Narod S.A. (2011). Frequencies of *BRCA1* and *BRCA2* Mutations among 1,342 Unselected Patients with Invasive Ovarian Cancer. Gynecol. Oncol..

[6] Levy-Lahad E., Friedman E. (2007). Cancer Risks among *BRCA1* and *BRCA2* Mutation Carriers. Br. J. Cancer.

[7] Rebbeck T.R., Mitra N., Wan F., Sinilnikova O.M., Healey S., McGuffog L., Mazoyer S., Chenevix-Trench G., Easton D.F., Antoniou A.C. (2015). Association of Type and Location of *BRCA1* and *BRCA2* Mutations with Risk of Breast and Ovarian Cancer. JAMA.

[8] Ramus S.J., Antoniou A.C., Kuchenbaecker K.B., Soucy P., Beesley J., Chen X., McGuffog L., Sinilnikova O.M., Healey S., Barrowdale D. (2012). Ovarian Cancer Susceptibility Alleles and Risk of Ovarian Cancer in *BRCA1* and *BRCA2* Mutation Carriers. Hum. Mutat..

[9] Gronwald J., Byrski T., Huzarski T., Oszurek O., Janicka A., Szymanska-Pasternak J., Górski B., Menkiszak J., Rzepka-Górska I., Lubinski J. (2008). Hereditary Breast and Ovarian Cancer. Hered. Cancer Clin. Pract..

[10] Friebel T.M., Domchek S.M., Rebbeck T.R. (2014). Modifiers of Cancer Risk in *BRCA1* and *BRCA2* Mutation Carriers: Systematic Review and Meta-Analysis. J. Natl. Cancer Inst..

[11] Brandao R.D. (2012). Improving the Risk Assessment of Inherited Breast and Ovarian Cancer: Clinical Significance of *BRCA1/2* Variants and Risk Modifiers. Ph.D. Thesis.

[12] Baquero J.M., Benítez-Buelga C., Fernández V., Urioste M., García-Giménez J.L., Perona R., Benítez J., Osorio A., The CIMBA Consortium (2019). A Common SNP in the UNG Gene Decreases Ovarian Cancer Risk in 2 Mutation Carriers. Mol. Oncol..

[13] Chenevix-Trench G., Milne R.L., Antoniou A.C., Couch F.J., Easton D.F., Goldgar D.E. (2007). An International Initiative to Identify Genetic Modifiers of Cancer Risk in *BRCA1* and *BRCA2* Mutation Carriers: The Consortium of Investigators of Modifiers of *BRCA1* and *BRCA2* (CIMBA). Breast Cancer Res..

[14] Bolton K.L., Ganda C., Berchuck A., Pharaoh P.D.P., Gayther S.A. (2012). Role of Common Genetic Variants in Ovarian Cancer Susceptibility and Outcome: Progress to Date from the Ovarian Cancer Association Consortium (OCAC). J. Intern. Med..

[15] Page M.J., McKenzie J.E., Bossuyt P.M., Boutron I., Hoffmann T.C., Mulrow C.D., Shamseer L., Tetzlaff J.M., Akl E.A., Brennan S.E. (2021). The PRISMA 2020 Statement: An Updated Guideline for Reporting Systematic Reviews. BMJ.

[16] Milne R.L., Antoniou A.C. (2016). Modifiers of Breast and Ovarian Cancer Risks for *BRCA1* and *BRCA2* Mutation Carriers. Endocr. Relat. Cancer.

[17] Pearce C.L., Hirschhorn J.N., Wu A.H., Burtt N.P., Stram D.O., Young S., Kolonel L.N., Henderson B.E., Altshuler D., Pike M.C. (2005). Clarifying the PROGINS Allele Association in Ovarian and Breast Cancer Risk: A Haplotype-Based Analysis. J. Natl. Cancer Inst..

[18] Doherty J.A., Rossing M.A., Cushing-Haugen K.L., Chen C., Van Den Berg D.J., Wu A.H., Pike M.C., Ness R.B., Moysich K., Chenevix-Trench G. (2010). ESR1/SYNE1 Polymorphism and Invasive Epithelial Ovarian Cancer Risk: An Ovarian Cancer Association Consortium Study. Cancer Epidemiol. Biomark. Prev. Publ..

[19] Kang S., Ju W., Kim J.W., Park N.-H., Song Y.-S., Kim S.C., Park S.-Y., Kang S.-B., Lee H.-P. (2006). Association between Excision Repair Cross-Complementation Group 1 Polymorphism and Clinical Outcome of Platinum-Based Chemotherapy in Patients with Epithelial Ovarian Cancer. Exp. Mol. Med..

[20] Gayther S.A., Song H., Ramus S.J., Kjaer S.K., Whittemore A.S., Quaye L., Tyrer J., Shadforth D., Hogdall E., Hogdall C. (2007). Tagging Single Nucleotide Polymorphisms in Cell Cycle Control Genes and Susceptibility to Invasive Epithelial Ovarian Cancer. Cancer Res..

[21] Cunningham J.M., Vierkant R.A., Sellers T.A., Phelan C., Rider D.N., Liebow M., Schildkraut J., Berchuck A., Couch F.J., Wang X. (2009). Cell Cycle Genes and Ovarian Cancer Susceptibility: A tagSNP Analysis. Br. J. Cancer.

[22] Quaye L., Song H., Ramus S.J., Gentry-Maharaj A., Høgdall E., DiCioccio R.A., McGuire V., Wu A.H., Van Den Berg D.J., Pike M.C. (2009). Tagging Single-Nucleotide Polymorphisms in Candidate Oncogenes and Susceptibility to Ovarian Cancer. Br. J. Cancer.

[23] Quaye L., Gayther S.A., Ramus S.J., Di Cioccio R.A., McGuire V., Hogdall E., Hogdall C., Blaakr J., Easton D.F., Ponder B.A.J. (2008). The Effects of Common Genetic Variants in Oncogenes on Ovarian Cancer Survival. Clin. Cancer Res. Off. J. Am. Assoc. Cancer Res..

[24] Schildkraut J.M., Goode E.L., Clyde M.A., Iversen E.S., Moorman P.G., Berchuck A., Marks J.R., Lissowska J., Brinton L., Peplonska B. (2009). Single Nucleotide Polymorphisms in the TP53 Region and Susceptibility to Invasive Epithelial Ovarian Cancer. Cancer Res..

[25] Song H., Ramus S.J., Shadforth D., Quaye L., Kjaer S.K., Dicioccio R.A., Dunning A.M., Hogdall E., Hogdall C., Whittemore A.S. (2006). Common Variants in RB1 Gene and Risk of Invasive Ovarian Cancer. Cancer Res..

[26] Uffelmann E., Huang Q.Q., Munung N.S., de Vries J., Okada Y., Martin A.R., Martin H.C., Lappalainen T., Posthuma D. (2021). Genome-Wide Association Studies. Nat. Rev. Methods Primers.

[27] Garcia-Closas M., Chanock S. (2008). Genetic Susceptibility Loci for Breast Cancer by Estrogen Receptor (ER) Status. Clin. Cancer Res. Off. J. Am. Assoc. Cancer Res..

[28] Dudbridge F., Gusnanto A. (2008). Estimation of Significance Thresholds for Genomewide Association Scans. Genet. Epidemiol..

[29] Song H., Ramus S.J., Tyrer J., Bolton K.L., Gentry-Maharaj A., Wozniak E., Anton-Culver H., Chang-Claude J., Cramer D.W., DiCioccio R. (2009). A Genome-Wide Association Study Identifies a New Ovarian Cancer Susceptibility Locus On 9p22.2. Nat. Genet..

[30] Pharoah P.D.P., Tsai Y.-Y., Ramus S.J., Phelan C.M., Goode E.L., Lawrenson K., Price M., Fridley B.L., Tyrer J.P., Shen H. (2013). GWAS Meta-Analysis and Replication Identifies Three New Susceptibility Loci for Ovarian Cancer. Nat. Genet..

[31] Permuth-Wey J., Lawrenson K., Shen H.C., Velkova A., Tyrer J.P., Chen Z., Lin H.-Y., Ann Chen Y., Tsai Y.-Y., Qu X. (2013). Identification and Molecular Characterization of a New Ovarian Cancer Susceptibility Locus at 17q21.31. Nat. Commun..

[32] Goode E.L., Chenevix-Trench G., Song H., Ramus S.J., Notaridou M., Lawrenson K., Widschwendter M., Vierkant R.A., Larson M.C., Kjaer S.K. (2010). A Genome-Wide Association Study Identifies Susceptibility Loci for Ovarian Cancer at 2q31 and 8q24. Nat. Genet..

[33] Bolton K.L., Tyrer J., Song H., Ramus S.J., Notaridou M., Jones C., Sher T., Gentry-Maharaj A., Wozniak E., Tsai Y.-Y. (2010). Common Variants at 19p13 Are Associated with Susceptibility to Ovarian Cancer. Nat. Genet..

[34] Manolio T.A. (2013). Bringing Genome-Wide Association Findings into Clinical Use. Nat. Rev. Genet..

[35] Manolio T.A., Collins F.S., Cox N.J., Goldstein D.B., Hindorff L.A., Hunter D.J., McCarthy M.I., Ramos E.M., Cardon L.R., Chakravarti A. (2009). Finding the Missing Heritability of Complex Diseases. Nature.

[36] Wei W.-H., Hemani G., Haley C.S. (2014). Detecting Epistasis in Human Complex Traits. Nat. Rev. Genet..

[37] Hindorff L.A., Sethupathy P., Junkins H.A., Ramos E.M., Mehta J.P., Collins F.S., Manolio T.A. (2009). Potential Etiologic and Functional Implications of Genome-Wide Association Loci for Human Diseases and Traits. Proc. Natl. Acad. Sci. USA.

[38] Tam V., Patel N., Turcotte M., Bossé Y., Paré G., Meyre D. (2019). Benefits and Limitations of Genome-Wide Association Studies. Nat. Rev. Genet..

[39] Lawrenson K., Song F., Hazelett D.J., Kar S.P., Tyrer J., Phelan C.M., Corona R.I., Rodríguez-Malavé N.I., Seo J.-H., Adler E. (2019). Genome-Wide Association Studies Identify Susceptibility Loci for Epithelial Ovarian Cancer in East Asian Women. Gynecol. Oncol..

[40] Ramus S.J., Kartsonaki C., Gayther S.A., Pharoah P.D.P., Sinilnikova O.M., Beesley J., Chen X., McGuffog L., Healey S., Couch F.J. (2011). Genetic Variation at 9p22.2 and Ovarian Cancer Risk for *BRCA1* and *BRCA2* Mutation Carriers. J. Natl. Cancer Inst..

[41] Kuchenbaecker K.B., Ramus S.J., Tyrer J., Lee A., Shen H.C., Beesley J., Lawrenson K., McGuffog L., Healey S., Lee J.M. (2015). Identification of Six New Susceptibility Loci for Invasive Epithelial Ovarian Cancer. Nat. Genet..

[42] Ioannidis J.P.A. (2008). Why Most Discovered True Associations Are Inflated. Epidemiology.

[43] Sayols-Baixeras S., Lluís-Ganella C., Lucas G., Elosua R. (2014). Pathogenesis of Coronary Artery Disease: Focus on Genetic Risk Factors and Identification of Genetic Variants. Appl. Clin. Genet..

[44] Frazier-Wood A.C. (2015). Dietary Patterns, Genes, and Health: Challenges and Obstacles to Be Overcome. Curr. Nutr. Rep..

[45] Pearson T.A., Manolio T.A. (2008). How to Interpret a Genome-Wide Association Study. JAMA.

[46] Hirschhorn J.N., Lohmueller K., Byrne E., Hirschhorn K. (2002). A Comprehensive Review of Genetic Association Studies. Genet. Med..

[47] Shabalin A.A., Aberg K.A. (2015). Candidate Gene Methylation Studies Are at High Risk of Erroneous Conclusions. Epigenomics.

[48] Osorio A., Milne R.L., Kuchenbaecker K., Vaclová T., Pita G., Alonso R., Peterlongo P., Blanco I., de la Hoya M., Duran M. (2014). DNA Glycosylases Involved in Base Excision Repair May Be Associated with Cancer Risk in *BRCA1* and *BRCA2* Mutation Carriers. PLoS Genet..

[49] Jakubowska A., Gronwald J., Menkiszak J., Górski B., Huzarski T., Byrski T., Edler L., Lubiński J., Scott R.J., Hamann U. (2007). Methylenetetrahydrofolate Reductase Polymorphisms Modify *BRCA1*-Associated Breast and Ovarian Cancer Risks. Breast Cancer Res. Treat..

[50] Pepe C., Guidugli L., Sensi E., Aretini P., D’Andrea E., Montagna M., Manoukian S., Ottini L., Radice P., Viel A. (2007). Methyl Group Metabolism Gene Polymorphisms as Modifier of Breast Cancer Risk in Italian *BRCA1/2* Carriers. Breast Cancer Res. Treat..

[51] Jakubowska A., Rozkrut D., Antoniou A., Hamann U., Scott R.J., McGuffog L., Healy S., Sinilnikova O.M., Rennert G., Lejbkowicz F. (2012). Association of PHB 1630 C>T and MTHFR 677 C>T Polymorphisms with Breast and Ovarian Cancer Risk in *BRCA1/2* Mutation Carriers: Results from a Multicenter Study. Br. J. Cancer.

[52] Jakubowska A., Gronwald J., Menkiszak J., Górski B., Huzarski T., Byrski T., Edler L., Lubiński J., Scott R.J., Hamann U. (2007). Integrin Β3 Leu33Pro Polymorphism Increases *BRCA1*-associated Ovarian Cancer Risk. J. Med. Genet..

[53] Jakubowska A., Rozkrut D., Antoniou A., Hamann U., Lubinski J. (2010). The Leu33Pro Polymorphism in the *ITGB3* Gene Does Not Modify *BRCA1/2*-Associated Breast or Ovarian Cancer Risks: Results from a Multicenter Study among 15,542 *BRCA1* and *BRCA2* Mutation Carriers. Breast Cancer Res. Treat..

[54] Dick M.G., Versmold B., Engel C., Meindl A., Arnold N., Varon-Mateeva R., Sutter C., Niederacher D., Deissler H., Preisler-Adams S. (2012). Association of Death Receptor 4 Variant (683A>C) with Ovarian Cancer Risk in *BRCA1* Mutation Carriers. Int. J. Cancer.

[55] Couch F.J., Wang X., McGuffog L., Lee A., Olswold C., Kuchenbaecker K.B., Soucy P., Fredericksen Z., Barrowdale D., Dennis J. (2013). Genome-Wide Association Study in *BRCA1* Mutation Carriers Identifies Novel Loci Associated with Breast and Ovarian Cancer Risk. PLoS Genet..

[56] Phelan C.M., Rebbeck T.R., Weber B.L., Devilee P., Ruttledge M.H., Lynch H.T., Lenoir G.M., Stratton M.R., Easton D.F., Ponder B.A.J. (1996). Ovarian Cancer Risk in *BRCA1* Carriers Is Modified by the *HRAS1* Variable Number of Tandem Repeat (VNTR) Locus. Nat. Genet..

[57] Yarden R.I., Friedman E., Metsuyanim S., Olender T., Ben-Asher E., Papa M.Z. (2008). MDM2 SNP309 Accelerates Breast and Ovarian Carcinogenesis in *BRCA1* and *BRCA2* Carriers of Jewish–Ashkenazi Descent. Breast Cancer Res. Treat..

[58] Bjørnslett M., Knappskog S., Lønning P.E., Dørum A. (2012). Effect of the MDM2 Promoter Polymorphisms SNP309T>G and SNP285G>C on the Risk of Ovarian Cancer in *BRCA1* Mutation Carriers. BMC Cancer.

[59] Walker L.C., Marquart L., Pearson J.F., Wiggins G.A.R., O’Mara T.A., Parsons M.T., BCFR, Barrowdale D., McGuffog L., Dennis J. (2017). Evaluation of Copy-Number Variants as Modifiers of Breast and Ovarian Cancer Risk for *BRCA1* Pathogenic Variant Carriers. Eur. J. Hum. Genet..

[60] Engel C., Versmold B., Wappenschmidt B., Simard J., Easton D.F., Peock S., Cook M., Oliver C., Frost D., Mayes R. (2010). Association of the Variants *CASP8* D302H and *CASP10* V410I with Breast and Ovarian Cancer Risk in *BRCA1* and *BRCA2* Mutation Carriers. Cancer Epidemiol. Biomark. Prev. Publ..

[61] Rebbeck T.R., Mitra N., Domchek S.M., Wan F., Chuai S., Friebel T.M., Panossian S., Spurdle A., Chenevix-Trench G., kConFab (2009). Modification of Ovarian Cancer Risk by *BRCA1/2*-Interacting Genes in a Multicenter Cohort of *BRCA1/2* Mutation Carriers. Cancer Res..

[62] López B., Miguel J. (2021). Functional Validation of DNA Glycosylases as Cancer Risk Modifiers in *BRCA1* and *BRCA2* Mutation Carriers. Potential Use of *OGG1* Inhibitors as a Novel Strategy for Cancer Treatment. Ph.D. Thesis.

[63] Vigorito E., Kuchenbaecker K.B., Beesley J., Adlard J., Agnarsson B.A., Andrulis I.L., Arun B.K., Barjhoux L., Belotti M., Benitez J. (2016). Fine-Scale Mapping at 9p22.2 Identifies Candidate Causal Variants That Modify Ovarian Cancer Risk in *BRCA1* and *BRCA2* Mutation Carriers. PLoS ONE.

[64] Yarmolinsky J., Bull C.J., Vincent E.E., Robinson J., Walther A., Smith G.D., Lewis S.J., Relton C.L., Martin R.M. (2020). Association Between Genetically Proxied Inhibition of HMG-CoA Reductase and Epithelial Ovarian Cancer. JAMA.

[65] Ding Y.C., McGuffog L., Healey S., Friedman E., Laitman Y., Paluch-Shimon S., Kaufman B., SWE-BRCA, Liljegren A., Lindblom A. (2012). A Nonsynonymous Polymorphism in *IRS1* Modifies Risk of Developing Breast and Ovarian Cancers in *BRCA1* and Ovarian Cancer in *BRCA2* Mutation Carriers. Cancer Epidemiol. Biomark. Prev..

[66] Couch F.J., Gaudet M.M., Antoniou A.C., Ramus S.J., Kuchenbaecker K.B., Soucy P., Beesley J., Chen X., Wang X., Kirchhoff T. (2012). Common Variants at the 19p13.1 and *ZNF365* Loci Are Associated with ER Subtypes of Breast Cancer and Ovarian Cancer Risk in *BRCA1* and *BRCA2* Mutation Carriers. Cancer Epidemiol. Biomark. Prev..

[67] Zhu Q., Wang J., Yu H., Hu Q., Bateman N.W., Long M., Rosario S., Schultz E., Dalgard C.L., Wilkerson M.D. (2022). Whole-Genome Sequencing Identifies *PPARGC1A* as a Putative Modifier of Cancer Risk in *BRCA1/2* Mutation Carriers. Cancers.

[68] Tomaselli S., Megiorni F., Lin L., Mazzilli M.C., Gerrelli D., Majore S., Grammatico P., Achermann J.C. (2011). Human *RSPO1*/R-Spondin1 Is Expressed during Early Ovary Development and Augments β-Catenin Signaling. PLoS ONE.

[69] Ruiz De Garibay G., Fernandez-Garcia I., Mazoyer S., Leme De Calais F., Ameri P., Vijayakumar S., Martinez-Ruiz H., Damiola F., Barjhoux L., Thomassen M. (2021). Altered Regulation of *BRCA1* Exon 11 Splicing Is Associated with Breast Cancer Risk in Carriers of *BRCA1* Pathogenic Variants. Hum. Mutat..

[70] Hughes D.J., Ginolhac S.M., Coupier I., Corbex M., Bressac-de-Paillerets B., Chompret A., Bignon Y.-J., Uhrhammer N., Lasset C., Giraud S. (2005). Common *BRCA2* Variants and Modification of Breast and Ovarian Cancer Risk in *BRCA1* Mutation Carriers. Cancer Epidemiol. Biomark. Prev..

[71] Jakubowska A., Gronwald J., Menkiszak J., Górski B., Huzarski T., Byrski T., Tołoczko-Grabarek A., Gilbert M., Edler L., Zapatka M. (2010). *BRCA1*-Associated Breast and Ovarian Cancer Risks in Poland: No Association with Commonly Studied Polymorphisms. Breast Cancer Res. Treat..

[72] Osorio A., Pollán M., Pita G., Schmutzler R.K., Versmold B., Engel C., Meindl A., Arnold N., Preisler-Adams S., Niederacher D. (2008). An Evaluation of the Polymorphisms Ins16bp and Arg72Pro in *P53* as Breast Cancer Risk Modifiers in *BRCA1* and *BRCA2* Mutation Carriers. Br. J. Cancer.

[73] Osorio A., Martínez-Delgado B., Pollán M., Cuadros M., Urioste M., Torrenteras C., Melchor L., Díez O., De La Hoya M., Velasco E. (2006). A Haplotype Containing the *p53* Polymorphisms Ins16bp and Arg72Pro Modifies Cancer Risk in *BRCA2* Mutation Carriers. Hum. Mutat..

[74] Wang-Gohrke S., Weikel W., Risch H., Vesprini D., Abrahamson J., Lerman C., Godwin A., Moslehi R., Olipade O., Brunet J.-S. (1999). Intron Variants of the *p53* Gene Are Associated with Increased Risk for Ovarian Cancer but Not in Carriers of *BRCA1* or *BRCA2* Germline Mutations. Br. J. Cancer.

[75] Antoniou A.C., Kartsonaki C., Sinilnikova O.M., Soucy P., McGuffog L., Healey S., Lee A., Peterlongo P., Manoukian S., Peissel B. (2011). Common Alleles at 6q25.1 and 1p11.2 Are Associated with Breast Cancer Risk for *BRCA1* and *BRCA2* Mutation Carriers. Hum. Mol. Genet..

[76] Given H.F., Radbourne R., Oag H., Merritt S., Barclay E., Hanby A.M., Lamlum H., McGrath J., Curran C., Tomlinson I.P.M. (2000). The Androgen Receptor Exon 1 Trinucleotide Repeat Does Not Act as a Modifier of the Age of Presentation in Breast Cancer. Eur. J. Cancer.

[77] Kadouri L., Easton D.F., Edwards S., Hubert A., Kote-Jarai Z., Glaser B., Durocher F., Abeliovich D., Peretz T., Eeles R.A. (2001). CAG and GGC Repeat Polymorphisms in the Androgen Receptor Gene and Breast Cancer Susceptibility in *BRCA1/2* Carriers and Non-Carriers. Br. J. Cancer.

[78] Menin C., Banna G.L., De Salvo G., Lazzarotto V., De Nicolo A., Agata S., Montagna M., Sordi G., Nicoletto O., Chieco-Bianchi L. (2001). Lack of Association between Androgen Receptor CAG Polymorphism and Familial Breast/Ovarian Cancer. Cancer Lett..

[79] Kim S.C., Ju W., Mahavni V., Geisler J.P., Buller R.E. (2006). CAG Repeat Length in Exon 1 of the Androgen Receptor Gene Is Related to Age of Diagnosis but Not Germ Line *BRCA1* Mutation Status in Ovarian Cancer. Int. J. Gynecol. Cancer.

[80] Li A.J., McAllister P., Karlan B.Y. (2010). Impact of Androgen Receptor Cytosine-Adenine-Guanine Polymorphisms on Clinical Outcome in *BRCA* Mutation-Associated Epithelial Ovarian Cancers. Gynecol. Oncol..

[81] Runnebaum I.B., Wang-Gohrke S., Vesprini D., Kreienberg R., Lynch H., Moslehi R., Ghadirian P., Weber B., Godwin A.K., Risch H. (2001). Progesterone Receptor Variant Increases Ovarian Cancer Risk in *BRCA1* and *BRCA2* Mutation Carriers Who Were Never Exposed to Oral Contraceptives. Pharmacogenetics.

[82] Jakubowska A., Gronwald J., Menkiszak J., Górski B., Huzarski T., Byrski T., Benner A., Lubiński J., Scott R.J., Hamann U. (2008). Ovarian Cancer Risk in Polish *BRCA1* Mutation Carriers Is Not Associated with the Prohibitin 3′ Untranslated Region Polymorphism. BMC Cancer.

[83] Fung W.L.A., Risch H., McLaughlin J., Rosen B., Cole D., Vesprini D., Narod S.A. (2003). The *N314D* Polymorphism of *Galactose-1-Phosphate Uridyl Transferase* Does Not Modify the Risk of Ovarian Cancer. Cancer Epidemiol. Biomark. Prev..

[84] Pharoah P.D.P., Palmieri R.T., Ramus S.J., Gayther S.A., Andrulis I.L., Anton-Culver H., Antonenkova N., Antoniou A.C., Goldgar D., Beattie M.S. (2011). The Role of *KRAS* Rs61764370 in Invasive Epithelial Ovarian Cancer: Implications for Clinical Testing. Clin. Cancer Res..

[85] Hollestelle A., van der Baan F.H., Berchuck A., Johnatty S.E., Aben K.K., Agnarsson B.A., Aittomäki K., Alducci E., Andrulis I.L., Ovarian Cancer Association Consortium, Breast Cancer Association Consortium, and Consortium of Modifiers of *BRCA1* and *BRCA2* (2016). No Clinical Utility of *KRAS* Variant Rs61764370 for Ovarian or Breast Cancer. Gynecol. Oncol..

[86] Laitman Y., Kuchenbaecker K.B., Rantala J., Hogervorst F., Peock S., Godwin A.K., Arason A., Kirchhoff T., Offit K., Isaacs C. (2012). The KL-VS Sequence Variant of Klotho and Cancer Risk in *BRCA1* and *BRCA2* Mutation Carriers. Breast Cancer Res. Treat..

[87] Pooley K.A., McGuffog L., Barrowdale D., Frost D., Ellis S.D., Fineberg E., Platte R., Izatt L., Adlard J., Bardwell J. (2014). Lymphocyte Telomere Length Is Long in *BRCA1* and *BRCA2* Mutation Carriers Regardless of Cancer-Affected Status. Cancer Epidemiol. Biomark. Prev..

[88] Aghmesheh M., Suo Z., Friedlander M., Nesland J.M., Kaern J., Stewart M., Kconfab, Dorum A., Tucker K.M., Buckley M.F. (2006). Chromosome 2q24.2 Is Lost in Sporadic but Not in *BRCA1*-Associated Ovarian Carcinomas. Pathology.

[89] Hamdi Y., Soucy P., Kuchenbaeker K.B., Pastinen T., Droit A., Lemaçon A., Adlard J., Aittomäki K., Andrulis I.L., Arason A. (2017). Association of Breast Cancer Risk in *BRCA1* and *BRCA2* Mutation Carriers with Genetic Variants Showing Differential Allelic Expression: Identification of a Modifier of Breast Cancer Risk at Locus 11q22.3. Breast Cancer Res. Treat..

[90] Gaudet M.M., Kuchenbaecker K.B., Vijai J., Klein R.J., Kirchhoff T., McGuffog L., Barrowdale D., Dunning A.M., Lee A., Dennis J. (2013). Identification of a *BRCA2*-Specific Modifier Locus at 6p24 Related to Breast Cancer Risk. PLoS Genet..

[91] Peterlongo P., Chang-Claude J., Moysich K.B., Rudolph A., Schmutzler R.K., Simard J., Soucy P., Eeles R.A., Easton D.F., Hamann U. (2015). Candidate Genetic Modifiers for Breast and Ovarian Cancer Risk in *BRCA1* and *BRCA2* Mutation Carriers. Cancer Epidemiol. Biomark. Prev..

[92] LeVasseur N., Chia S. (2019). Cancer Screening and Prevention in *BRCA* Mutation Carriers: A Missed Opportunity?. Br. J. Cancer.

[93] Parker W.H., Jacoby V., Shoupe D., Rocca W. (2009). Effect of Bilateral Oophorectomy on Women’s Long-Term Health. Women’s Health.

[94] Rivera C.M., Grossardt B.R., Rhodes D.J., Rocca W.A. (2009). Increased Mortality for Neurological and Mental Diseases Following Early Bilateral Oophorectomy. Neuroepidemiology.

[95] Kuchenbaecker K.B., McGuffog L., Barrowdale D., Lee A., Soucy P., Healey S., Dennis J., Lush M., Robson M., Spurdle A.B. (2017). Evaluation of Polygenic Risk Scores for Breast and Ovarian Cancer Risk Prediction in *BRCA1* and *BRCA2* Mutation Carriers. J. Natl. Cancer Inst..

[96] Barnes D.R., Rookus M.A., McGuffog L., Leslie G., Mooij T.M., Dennis J., Mavaddat N., Adlard J., Ahmed M., Aittomäki K. (2020). Polygenic Risk Scores and Breast and Epithelial Ovarian Cancer Risks for Carriers of *BRCA1* and *BRCA2* Pathogenic Variants. Genet. Med..

[97] Dareng E.O., Tyrer J.P., Barnes D.R., Jones M.R., Yang X., Aben K.K.H., Adank M.A., Agata S., Andrulis I.L., Anton-Culver H. (2022). Polygenic Risk Modeling for Prediction of Epithelial Ovarian Cancer Risk. Eur. J. Hum. Genet..

[98] Yang X., Leslie G., Gentry-Maharaj A., Ryan A., Intermaggio M., Lee A., Kalsi J.K., Tyrer J., Gaba F., Manchanda R. (2018). Evaluation of Polygenic Risk Scores for Ovarian Cancer Risk Prediction in a Prospective Cohort Study. J. Med. Genet..

[99] Bogdan R., Baranger D.A.A., Agrawal A. (2018). Polygenic Risk Scores in Clinical Psychology: Bridging Genomic Risk to Individual Differences. Annu. Rev. Clin. Psychol..

[100] Carver T., Hartley S., Lee A., Cunningham A.P., Archer S., Babb de Villiers C., Roberts J., Ruston R., Walter F.M., Tischkowitz M. (2020). CanRisk Tool—A Web Interface for the Prediction of Breast and Ovarian Cancer Risk and the Likelihood of Carrying Genetic Pathogenic Variants. Cancer Epidemiol. Biomark. Prev..

[101] Møller N.B., Boonen D.S., Feldner E.S., Hao Q., Larsen M., Lænkholm A.-V., Borg Å., Kvist A., Törngren T., Jensen U.B. (2023). Validation of the BOADICEA Model for Predicting the Likelihood of Carrying Pathogenic Variants in Eight Breast and Ovarian Cancer Susceptibility Genes. Sci. Rep..

[102] Yang X., Wu Y., Ficorella L., Wilcox N., Dennis J., Tyrer J., Carver T., Pashayan N., Tischkowitz M., Pharoah P.D. (2024). Validation of the BOADICEA Model for Epithelial Tubo-Ovarian Cancer Risk Prediction in UK Biobank. Br. J. Cancer.

[103] Phillips S., Landy R., Bodelon C., Machiela M.J., Temkin S.M., Wentzensen N. (2024). Association of a Polygenic Risk Score with Risk of Abnormal Ultrasound Findings and Ovarian Cancer in the Prostate, Lung, Colorectal, and Ovarian Cancer Screening Trial. J. Clin. Oncol..

[104] Lewis C.M., Vassos E. (2020). Polygenic Risk Scores: From Research Tools to Clinical Instruments. Genome Med..

[105] Ottman R. (1996). Gene–Environment Interaction: Definitions and Study Designs. Prev. Med..

[106] Kim M.S., Patel K.P., Teng A.K., Berens A.J., Lachance J. (2018). Genetic Disease Risks Can Be Misestimated across Global Populations. Genome Biol..

[107] Wang Y., Zhu M., Ma H., Shen H. (2021). Polygenic Risk Scores: The Future of Cancer Risk Prediction, Screening, and Precision Prevention. Med. Rev..

[108] Skinner C.S., Schildkraut J.M., Berry D., Calingaert B., Marcom P.K., Sugarman J., Winer E.P., Iglehart J.D., Futreal P.A., Rimer B.K. (2002). Pre-Counseling Education Materials for *BRCA* Testing: Does Tailoring Make a Difference?. Genet. Test..

[109] Barnes D.R., Antoniou A.C. (2012). Unravelling Modifiers of Breast and Ovarian Cancer Risk for *BRCA1* and *BRCA2* Mutation Carriers: Update on Genetic Modifiers. J. Intern. Med..

[110] Chan J.L., Johnson L.N., Sammel M.D., DiGiovanni L., Voong C., Domchek S.M., Gracia C.R. (2017). Reproductive Decision-Making in Women with *BRCA1/2* Mutations. J. Genet. Couns..

[111] Hesse-Biber S., Seven M., Jiang J., Van Schaik S., Dwyer A.A. (2022). Impact of *BRCA* Status on Reproductive Decision-Making and Self-Concept: A Mixed-Methods Study Informing the Development of Tailored Interventions. Cancers.

[112] Pal T., Permuth-Wey J., Betts J.A., Krischer J.P., Fiorica J., Arango H., LaPolla J., Hoffman M., Martino M.A., Wakeley K. (2005). *BRCA1* and *BRCA2* Mutations Account for a Large Proportion of Ovarian Carcinoma Cases. Cancer.

[113] Wentzensen N., Ring K., Erickson B.K., Reid B., O’Donnell T., Check D., Tworoger S.S., Choudhury P.P. (2025). Ovarian Cancer Risk Prediction: A Clinical Epidemiology Perspective. Am. J. Epidemiol..

[114] Kim S., Wang M., Tyrer J.P., Jensen A., Wiensch A., Liu G., Lee A.W., Ness R.B., Salvatore M., Tworoger S.S. (2019). A Comprehensive Gene-Environment Interaction Analysis in Ovarian Cancer Using Genome-Wide Significant Common Variants. Int. J. Cancer.

[115] Turnbull C., Seal S., Renwick A., Warren-Perry M., Hughes D., Elliott A., Pernet D., Peock S., Adlard J.W., Barwell J. (2012). Gene–Gene Interactions in Breast Cancer Susceptibility. Hum. Mol. Genet..

[116] Cano-Gamez E., Trynka G. (2020). From GWAS to Function: Using Functional Genomics to Identify the Mechanisms Underlying Complex Diseases. Front. Genet..

[117] Moffitt L.R., Karimnia N., Wilson A.L., Stephens A.N., Ho G.-Y., Bilandzic M. (2024). Challenges in Implementing Comprehensive Precision Medicine Screening for Ovarian Cancer. Curr. Oncol..

[118] Masoodi T., Siraj S., Siraj A.K., Azam S., Qadri Z., Parvathareddy S.K., Tulbah A., Al-Dayel F., AlHusaini H., AlOmar O. (2020). Genetic Heterogeneity and Evolutionary History of High-Grade Ovarian Carcinoma and Matched Distant Metastases. Br. J. Cancer.

[119] Kim S., Han Y., Kim S.I., Kim H.-S., Kim S.J., Song Y.S. (2018). Tumor Evolution and Chemoresistance in Ovarian Cancer. npj Precis. Oncol..

