# Supplementary material for: The Contribution of Genetic Modifiers to Ovarian Cancer Risk in BRCA1 and BRCA2 Pathogenic Variant Carriers
Source: Cancers (Basel). 2026 Jan 23;18(3):354. doi: 10.3390/cancers18030354 (PMC12897243; doi:10.3390/cancers18030354)
Supplement: Supplementary file 1 [file cancers-18-00354-s001.zip › cancers-4075866-supplementary.pdf]

Table S1. Polymorphisms not significantly associated with ovarian cancer risk in *BRCA1*/*BRCA2* PV carriers.

Gene	Locus	Polymorphism	Sample size	Unaffected/affected	OR/HR (95% CI)	*P*-value	Genotyping platform	Function	Ref
BRCA1	17q21.31	rs5820483	15 238	–	–	–	iCOGS	DNA repair	(7)
BRCA2	13q13.1	rs144848	778	–	–	–	Taqman	Homologous recombination	(8)
BRCA2	13q13.1	rs1799943	778	–	–	–	Taqman	Homologous recombination	(8)
TP53	17p13.1	237ins16 / c.441G>C / c.1798G>A	755	146/609	OR crude ~0.9–1.2	p (crude) >0.2	PCR-RFLP	Cell cycle control	(9-12)
RAD51L1	14q24.1	rs999737	Sample size not reported separately for ovarian cancer; ovarian and breast cancer analyzed jointly using a competing risks model		HR: 0.97 (0.86-1.10)	0.65	Illumina GWAS arrays	DNA repair	(13)
RAD51L1	14q24.1	rs10483813	Sample size not reported separately for ovarian cancer; ovarian and breast cancer analyzed jointly using a competing risks model		HR: 0.98 (0.87-1.11)	0.74	Illumina GWAS arrays	DNA repair	(13)
ERCC2	19q13.32	6540G>A	755	146/609	OR crude: ~0.8-1.0	p (crude) >0.3	PCR-RFLP	Nucleotide excision repair	(9)
XRCC1	19q13.32	rs25487	755	146/609	OR crude: ~1.0-1.2	p (crude) >0.3	PCR-RFLP	Base excision repair	(9)
XRCC2	7q36.1	27158G>A	755	146/609	OR crude: ~0.8-1.1	p (crude) >0.4	PCR-RFLP	Homologous recombination	(9)
XRCC3	14q32.33	rs861539	755	146/609	OR crude: **2.24** (1.02–4.92)	p (crude) ~0.04	PCR–	Homologous recombination	(9)
LIG4	13q33.3	rs1805388	755	146/609	OR crude: ~0.9-1.1	p (crude) >0.4	PCR-RFLP	Non-homologous end joining	(9)
AR	Xq11-12	CAG repeats	–	–	–	–	–	Steroid hormone signaling	(14-16)
AR	Xq11-12	GGC repeats	–	–	–	–	–	Steroid hormone signaling	(14-16)
PGR	11q22.1	PROGINS	778	583/195	OR: 1.11 (0.75 - 1.63)	NS	PCR	Progesterone receptor	(17)
PGR	11q22.1	rs10895068	755	146/609	OR crude: 1.55 (0.81-2.98)	p (crude): 0.18	PCR	Progesterone receptor signaling	(1)
PGR	11q22.1	88022ins306	755	146/609	OR crude: 1.43 (0.72-2.87)	p (crude): 0.32	PCR	Progesterone receptor signaling	(9)
ESR1	6q25.1	rs2046210	Sample size not reported separately for ovarian cancer; ovarian and breast cancer analyzed jointly using a competing risks model		HR: 1.05 (0.91-1.21)	0.50	Illumina GWAS arrays	Estrogen receptor alpha	(13)

ESR1	6q25.1	rs9397435	Sample size not reported separately for ovarian cancer; ovarian and breast cancer analyzed jointly using a competing risks model		HR: 1.03 (0.89-1.19)	0.67	Illumina GWAS arrays	Regulation of ESR1 expression	(13)
MTHFR	1p36.22	rs1801133	586	484/102	HR: 1.07 (0.63-1.82)	0.79	PCR	Folate metabolism	(2-4)
MTHFR	1p36.22	rs776842392	586	484/102	HR: 0.93 (0.55-1.60)	0.81	PCR	Folate metabolism	(3)
MS (MTR)	1q43	rs1805087	586	484/102	HR: 1.10 (0.63-1.91)	0.73	PCR-RFLP	Methionine metabolism	(3)
PHB	17q21	c.1630C>T	6195	5366/829	HR *BRCA1:* CT vs CC: 0.99 (0.82-1.21); TT vs CC: 0.80 (0.53-1.20) HR BRCA2: CT vs CC: 0.93; TT vs CC:0.92 (0.41-2.06)	*BRCA1:* CT vs CC: 0.91; TT vs CC: 0.28 BRCA2: CT vs CC:0.71; TT vs CC: 0.85	PCR-RFLP	Cell cycle regulation	(4)
ITGB3	17q21.32	rs5918	9998 *BRCA1* / 5544 *BRCA2*	8374/1624 *BRCA1* 5118/426 *BRCA2*	HR *BRCA1:* 1.11 (1.00-1.23), *BRCA2*: 1.09 (0.89-1.32)	*BRCA1:* 0.05, *BRCA2*: 0.38	Taqman	Mediates cell adhesion, signaling, and platelet aggregation	(5,6)
GALT	9p13.3	rs2070074	1183	364/891	OR: 0.94 (0.68-1.3) – for one allele 1.62 (0.34 – 7.7) – for two allele	0.70 – for one allele 0.54 – for two allele	Taqman	Galactose metabolism	(18)
TYMS	18p11.32	VNTR	755	146/609	OR crude ~0.9-1.1	p (crude) >0.5	PCR	Thymidylate synthesis	(9)
COMT	22q11.21	rs4680	755	146/609	OR crude: 1.85 (1.01-3.48)	p (crude) 0.04*	PCR-RFLP	Estrogen metabolism	(9)
CYP11A1	15q24.1	(AAAAT)n	755	146/609	OR crude: 0.54 (0.31- 0.95)	p (crude) ~0.03	PCR	Steroidogenesis initiation	(9)
CYP17A1	10q24.32	rs743572	755	146/609	OR crude: ~1.0 -1.1	p (crude) >0.3	PCR-RFLP	Steroid hormone biosynthesis	(9)
CYP19A1	15q21.2	15210(TTTA)n	755	146/609	OR crude: ~0.8-1.1	p (crude) >0.3	PCR	Aromatase / estrogen synthesis	(9)
HSD17	17q21.2	rs605059	755	146/609	OR crude: ~1.0-1.1	p (crude) >0.3	PCR-RFLP	Estrone estradiol conversion	(9)
KRAS	12p12.1	rs61764370	14 765 (BRCA1), 7 904 (BRCA2), 15 357 cases / 30 816 controls	–	HR: 1.09 (0.97-1.23) BRCA1; 0.89 (0.71-1.13) BRCA2, OR: 0.99 (0.94-1.04) OCAC	0.14 BRCA1, 0.34 BRCA2, 0.74 OCAC	iCOGS	GTPase signaling / cell proliferation and survival	(19,20)
KL (Klotho)	13q12.11	rs9536314 (KL-VS)	9080 (5741 BRCA1,	–	HR = 1.01 (0.84-1.20)	0.95 BRCA1,	iCOGS	Modulates endocrine	(21)

			3339 BRCA2)		BRCA1, 0.90 (0.66-1.22) BRCA2	0,49 BRCA2		signaling and cellular stress responses	
TERT	5p15.33	–	3682	–	–	–	–	Telomere maintenance	(22)
–	2q24.2	D2S156	21	0/21	–	0,385	Taqman	Microsatellite marker	(23)
ALP	1p36.12	rs12025623	1585	1300/285	HR: 1.098 (1.027-1.173)*	7×10^{-3}*	iCOGS	Encodes alkaline phosphatase important for bone mineralization	(24)
ALP	1p36.12	rs1767429	1585	1300/285	HR: 1.092 (1.024-1.164)*	9×10^{-3}*	iCOGS	Encodes alkaline phosphatase important for bone mineralization	(24)
MAD2L2	1p32.22	rs2233025	1350	1100/250	HR: 0.777 (0.657-0.919)*	5×10^{-3}*	iCOGS	Controls chromosome segregation and DNA damage response	(24)
AIB1	20q12	–	2383	1105/1278	–	> 0.05	iCOGS	Nuclear receptor co-activator influencing hormone signaling	(25)
HER2	17q12	–	2383	1105/1278	–	> 0.05	iCOGS	Receptor tyrosine kinase regulating cell proliferation	(25)
IL6	7p15.3	–	2383	1105/1278	–	> 0.05	iCOGS	Proinflammatory cytokine influencing immune responses	(25)
LRP1	12q13.3	–	2383	1105/1278	–	> 0.05	iCOGS	Endocytic receptor involved in cell signaling pathways	(25)
TGFB	19q13.2	–	2383	1105 /1278	–	> 0.05	iCOGS	TGF-β signalling, regulating cell growth and differentiation	(25)
TGFBR1	9q22.33	–	2383	1105 /1278	–	> 0.05	iCOGS	TGF-β signal transduction controlling proliferation	(25)

								and differentiation	
NOTCH2	1p12	–	2383	1105 /1278	–	> 0.05	iCOGS	Notch pathway receptor regulating differentiation	(25)
FGF13	Xq26.3	–	2383	1105 /1278	–	> 0.05	iCOGS	Member of fibroblast growth factor family (cell growth / migration)	(25)
6p24 locus	6p24	rs9348512	2383	1105 /1278	–	≈ 0.01–0.05	iCOGS	Regulatory locus near *TFAP2A* (transcription factor)	(25)
2p22 locus	2p22	rs184577	2383	1105 /1278	–	≈ 0.02–0.05)	iCOGS	Regulatory / *CYP1B1-AS1* region	(25)

* Nominal, before multiple testing

1. Siegel, R.L.; Miller, K.D.; Jemal, A. Cancer Statistics, 2020. *CA Cancer J. Clin.* **2020**, *70*, 7–30. https://doi.org/10.3322/caac.21590.
2. Jakubowska, A.; Gronwald, J.; Menkiszak, J.; Górski, B.; Huzarski, T.; Byrski, T.; Edler, L.; Lubiński, J.; Scott, R.J.; Hamann, U. Methylenetetrahydrofolate Reductase Polymorphisms Modify *BRCA1*-Associated Breast and Ovarian Cancer Risks. *Breast Cancer Res. Treat.* **2007**, *104*, 299–308. https://doi.org/10.1007/s10549-006-9417-3.
3. Pepe, C.; Guidugli, L.; Sensi, E.; Aretini, P.; D'Andrea, E.; Montagna, M.; Manoukian, S.; Ottini, L.; Radice, P.; Viel, A.; et al. Methyl Group Metabolism Gene Polymorphisms as Modifier of Breast Cancer Risk in Italian *BRCA1/2* Carriers. *Breast Cancer Res. Treat.* **2007**, *103*, 29–36. https://doi.org/10.1007/s10549-006-9349-y.
4. Jakubowska, A.; Rozkrut, D.; Antoniou, A.; Hamann, U.; Scott, R.J.; McGuffog, L.; Healy, S.; Sinilnikova, O.M.; Rennert, G.; Lejbkowicz, F.; et al. Association of PHB 1630 C>T and MTHFR 677 C>T Polymorphisms with Breast and Ovarian Cancer Risk in *BRCA1/2* Mutation Carriers: Results from a Multicenter Study. *Br. J. Cancer* **2012**, *106*, 2016–2024. https://doi.org/10.1038/bjc.2012.160.
5. Jakubowska, A.; Gronwald, J.; Menkiszak, J.; Górski, B.; Huzarski, T.; Byrski, T.; Edler, L.; Lubiński, J.; Scott, R.J.; Hamann, U. Integrin B3 Leu33Pro Polymorphism Increases *BRCA1*-associated Ovarian Cancer Risk. *J. Med. Genet.* **2007**, *44*, 408–411. https://doi.org/10.1136/jmg.2006.047498.
6. Jakubowska, A.; Rozkrut, D.; Antoniou, A.; Hamann, U.; Lubinski, J. The Leu33Pro Polymorphism in the *ITGB3* Gene Does Not Modify *BRCA1/2*-Associated Breast or Ovarian Cancer Risks: Results from a Multicenter Study among 15,542 *BRCA1* and *BRCA2* Mutation Carriers. *Breast Cancer Res. Treat.* **2010**, *121*, 639–649. https://doi.org/10.1007/s10549-009-0595-7.
7. Ruiz De Garibay, G.; Fernandez-Garcia, I.; Mazoyer, S.; Leme De Calais, F.; Ameri, P.; Vijayakumar, S.; Martinez-Ruiz, H.; Damiola, F.; Barjhoux, L.; Thomassen, M.; et al. Altered Regulation of *BRCA1* Exon 11 Splicing Is Associated with Breast Cancer Risk in Carriers of *BRCA1* Pathogenic Variants. *Hum. Mutat.* **2021**, *42*, 1488–1502. https://doi.org/10.1002/humu.24276.
8. Hughes, D.J.; Ginolhac, S.M.; Coupier, I.; Corbex, M.; Bressac-de-Paillerets, B.; Chompret, A.; Bignon, Y.-J.; Uhrhammer, N.; Lasset, C.; Giraud, S.; et al. Common *BRCA2* Variants and Modification of Breast and Ovarian Cancer Risk in *BRCA1* Mutation Carriers. *Cancer Epidemiol. Biomark. Prev.* **2005**, *14*, 265–267.
9. Jakubowska, A.; Gronwald, J.; Menkiszak, J.; Górski, B.; Huzarski, T.; Byrski, T.; Tołoczko-Grabarek, A.; Gilbert, M.; Edler, L.; Zapatka, M.; et al. *BRCA1*-Associated Breast and Ovarian Cancer Risks in Poland: No

Association with Commonly Studied Polymorphisms. *Breast Cancer Res. Treat.* **2010**, *119*, 201–211. https://doi.org/10.1007/s10549-009-0390-5.

10. Osorio, A.; Pollán, M.; Pita, G.; Schmutzler, R.K.; Versmold, B.; Engel, C.; Meindl, A.; Arnold, N.; Preisler-Adams, S.; Niederacher, D.; et al. An Evaluation of the Polymorphisms Ins16bp and Arg72Pro in *P53* as Breast Cancer Risk Modifiers in *BRCA1* and *BRCA2* Mutation Carriers. *Br. J. Cancer* **2008**, *99*, 974–977. https://doi.org/10.1038/sj.bjc.6604624.
11. Osorio, A.; Martínez-Delgado, B.; Pollán, M.; Cuadros, M.; Urioste, M.; Torrenteras, C.; Melchor, L.; Díez, O.; De La Hoya, M.; Velasco, E.; et al. A Haplotype Containing the *p53* Polymorphisms Ins16bp and Arg72Pro Modifies Cancer Risk in *BRCA2* Mutation Carriers. *Hum. Mutat.* **2006**, *27*, 242–248. https://doi.org/10.1002/humu.20283.
12. Wang-Gohrke, S.; Weikel, W.; Risch, H.; Vesprini, D.; Abrahamson, J.; Lerman, C.; Godwin, A.; Moslehi, R.; Olipade, O.; Brunet, J.-S.; et al. Intron Variants of the *p53* Gene Are Associated with Increased Risk for Ovarian Cancer but Not in Carriers of *BRCA1* or *BRCA2* Germline Mutations. *Br. J. Cancer* **1999**, *81*, 179–183. https://doi.org/10.1038/sj.bjc.6690669
13. Antoniou, A.C.; Kartsonaki, C.; Sinilnikova, O.M.; Soucy, P.; McGuffog, L.; Healey, S.; Lee, A.; Peterlongo, P.; Manoukian, S.; Peissel, B.; et al. Common Alleles at 6q25.1 and 1p11.2 Are Associated with Breast Cancer Risk for *BRCA1* and *BRCA2* Mutation Carriers. *Hum. Mol. Genet.* **2011**, *20*, 3304–3321. https://doi.org/10.1093/hmg/ddr226.
14. Given, H.F.; Radbourne, R.; Oag, H.; Merritt, S.; Barclay, E.; Hanby, A.M.; Lamlum, H.; McGrath, J.; Curran, C.; Tomlinson, I.P.M. The Androgen Receptor Exon 1 Trinucleotide Repeat Does Not Act as a Modifier of the Age of Presentation in Breast Cancer. *Eur. J. Cancer* **2000**, *36*, 533–534. https://doi.org/10.1016/S0959-8049(99)00310-X.
15. Kadouri, L.; Easton, D.F.; Edwards, S.; Hubert, A.; Kote-Jarai, Z.; Glaser, B.; Durocher, F.; Abeliovich, D.; Peretz, T.; Eeles, R.A. CAG and GGC Repeat Polymorphisms in the Androgen Receptor Gene and Breast Cancer Susceptibility in *BRCA1/2* Carriers and Non-Carriers. *Br. J. Cancer* **2001**, *85*, 36–40. https://doi.org/10.1054/bjoc.2001.1777.
16. Menin, C.; Banna, G.L.; De Salvo, G.; Lazzarotto, V.; De Nicolo, A.; Agata, S.; Montagna, M.; Sordi, G.; Nicoletto, O.; Chieco-Bianchi, L.; et al. Lack of Association between Androgen Receptor CAG Polymorphism and Familial Breast/Ovarian Cancer. *Cancer Lett.* **2001**, *168*, 31–36. https://doi.org/10.1016/S0304-3835(01)00473-6.
17. Runnebaum, I.B.; Wang-Gohrke, S.; Vesprini, D.; Kreienberg, R.; Lynch, H.; Moslehi, R.; Ghadirian, P.; Weber, B.; Godwin, A.K.; Risch, H.; et al. Progesterone Receptor Variant Increases Ovarian Cancer Risk in *BRCA1* and *BRCA2* Mutation Carriers Who Were Never Exposed to Oral Contraceptives. *Pharmacogenetics* **2001**, *11*, 635–638. https://doi.org/10.1097/00008571-200110000-00010.
18. Fung, W.L.A.; Risch, H.; McLaughlin, J.; Rosen, B.; Cole, D.; Vesprini, D.; Narod, S.A. The *N314D* Polymorphism of *Galactose-1-Phosphate Uridyl Transferase* Does Not Modify the Risk of Ovarian Cancer. *Cancer Epidemiol. Biomark. Prev.* **2003**, *12*, 678–680.
19. Pharoah, P.D.P.; Palmieri, R.T.; Ramus, S.J.; Gayther, S.A.; Andrulis, I.L.; Anton-Culver, H.; Antonenkova, N.; Antoniou, A.C.; Goldgar, D.; Beattie, M.S.; et al. The Role of *KRAS* Rs61764370 in Invasive Epithelial Ovarian Cancer: Implications for Clinical Testing. *Clin. Cancer Res.* **2011**, *17*, 3742–3750. https://doi.org/10.1158/1078-0432.CCR-10-3405.
20. Ovarian Cancer Association Consortium, Breast Cancer Association Consortium, and Consortium of Modifiers of *BRCA1* and *BRCA2*; Hollestelle, A.; van der Baan, F.H.; Berchuck, A.; Johnatty, S.E.; Aben, K.K.; Agnarsson, B.A.; Aittomäki, K.; Alducci, E.; Andrulis, I.L.; et al. No Clinical Utility of *KRAS* Variant Rs61764370 for Ovarian or Breast Cancer. *Gynecol. Oncol.* **2016**, *141*, 386–401. https://doi.org/10.1016/j.ygyno.2015.04.034.
21. Laitman, Y.; Kuchenbaecker, K.B.; Rantala, J.; Hogervorst, F.; Peock, S.; Godwin, A.K.; Arason, A.; Kirchhoff, T.; Offit, K.; Isaacs, C.; et al. The KL-VS Sequence Variant of Klotho and Cancer Risk in *BRCA1* and *BRCA2* Mutation Carriers. *Breast Cancer Res. Treat.* **2012**, *132*, 1119–1126. https://doi.org/10.1007/s10549-011-1938-8.

22. Pooley, K.A.; McGuffog, L.; Barrowdale, D.; Frost, D.; Ellis, S.D.; Fineberg, E.; Platte, R.; Izatt, L.; Adlard, J.; Bardwell, J.; et al. Lymphocyte Telomere Length Is Long in *BRCA1* and *BRCA2* Mutation Carriers Regardless of Cancer-Affected Status. *Cancer Epidemiol. Biomark. Prev.* **2014**, *23*, 1018–1024. https://doi.org/10.1158/1055-9965.EPI-13-0635-T.
23. Aghmesheh, M.; Suo, Z.; Friedlander, M.; Nesland, J.M.; Kaern, J.; Stewart, M.; Kconfab; Dorum, A.; Tucker, K.M.; Buckley, M.F. Chromosome 2q24.2 Is Lost in Sporadic but Not in *BRCA1*-Associated Ovarian Carcinomas. *Pathology* **2006**, *38*, 145–151. https://doi.org/10.1080/00313020600561526.
24. Hamdi, Y.; Soucy, P.; Kuchenbaeker, K.B.; Pastinen, T.; Droit, A.; Lemaçon, A.; Adlard, J.; Aittomäki, K.; Andrulis, I.L.; Arason, A.; et al. Association of Breast Cancer Risk in *BRCA1* and *BRCA2* Mutation Carriers with Genetic Variants Showing Differential Allelic Expression: Identification of a Modifier of Breast Cancer Risk at Locus 11q22.3. *Breast Cancer Res. Treat.* **2017**, *161*, 117–134. https://doi.org/10.1007/s10549-016-4018-2.
25. Gaudet, M.M.; Kuchenbaecker, K.B.; Vijai, J.; Klein, R.J.; Kirchhoff, T.; McGuffog, L.; Barrowdale, D.; Dunning, A.M.; Lee, A.; Dennis, J.; et al. Identification of a *BRCA2*-Specific Modifier Locus at 6p24 Related to Breast Cancer Risk. *PLoS Genet.* **2013**, *9*, e1003173. https://doi.org/10.1371/journal.pgen.1003173.
